# Time Scale Calculus: a new approach to multi-dose pharmacokinetic modeling

**DOI:** 10.1007/s10928-024-09920-z

**Published:** 2024-07-25

**Authors:** José Ricardo Arteaga-Bejarano, Santiago Torres

**Affiliations:** 1 Mathematics Department and Research Group in Mathematical and Computational Biology (BIOMAC), Universidad de los Andes, Cra 1 No 18A - 12, Bogotá, Colombia; 2 The Pearson Institute, Harris School of Public Policy, University of Chicago, 1307 East 60th Street, Chicago, USA

**Keywords:** Time Scale Calculus, Multi-dosing, Pharmacokinetic Models, Physiologically-based pharmacokinetic models, 26E70, 34N05, 39A60

## Abstract

In this paper, we use Time Scale Calculus (TSC) to formulate and solve pharmacokinetic models exploring multiple dose dynamics. TSC is a mathematical framework that allows the modeling of dynamical systems comprising continuous and discrete processes. This characteristic makes TSC particularly suited for multi-dose pharmacokinetic problems, which inherently feature a blend of continuous processes (such as absorption, metabolization, and elimination) and discrete events (drug intake). We use this toolkit to derive analytical expressions for blood concentration trajectories under various multi-dose regimens across several flagship pharmacokinetic models. We demonstrate that this mathematical framework furnishes an alternative and simplified way to model and retrieve analytical solutions for multi-dose dynamics. For instance, it enables the study of blood concentration responses to arbitrary dose regimens and facilitates the characterization of the long-term behavior of the solutions, such as their steady state.

**Supplementary Information:**

The online version contains supplementary material available at 10.1007/s10928-024-09920-z.

## Introduction

Time Scale Calculus (TSC) is an emerging field in mathematics that aims to unify the theory of differential and difference equations. While differential and difference equations require modeling time as exclusively a continuous or discrete quantity, TSC considers dynamics happening on unrestricted time domains called time scales. Hence, the core innovation of TSC lies in its capacity to simultaneously address continuous and discrete dynamics, offering an improved modeling framework for analyzing systems where these processes coexist. This approach was first introduced in the seminal works of [1, 2] and has attracted growing interest for its applicability across multiple disciplines.

This paper highlights the potential applications of TSC in Pharmacokinetic/Pharmacodynamic (PKPD) and Physiologically Based Pharmacokinetic (PBPK) modeling. In particular, we study blood concentration dynamics resulting from multiple administrations of orally and intravenously administered drugs. Understanding these dynamics is crucial for medical practice, as drugs are typically administered in a series of repetitive doses, not in single doses. TSC proves invaluable in examining these dynamics, offering a versatile framework that naturally accommodates the dual nature of drug administration: the continuous processes of absorption, metabolization, and elimination, alongside the discrete events of drug intake. Consequently, TSC provides a coherent language for integrating these processes into models, enhancing their interpretability, and expanding their applications and complexity.

The primary application examined herein is modeling multiple-dose dynamics through the Bateman function [3]. The Bateman function, which articulates the dynamics following some orally administered single doses, emerges from the solution of a second-order linear ordinary differential equation or, equivalently, from a system of two first-order linear equations, that rationalize well-known physiological processes. Thus, to extend this model to accommodate multiple doses, we employ TSC to transition from ordinary linear equations to dynamic equations—their generalization within the TSC framework—enabling the incorporation of dosing timings into the dynamics. This methodology demonstrates how TSC facilitates the derivation of analytical solutions for established problems, such as the “Equi-dosing” regime—wherein a constant drug quantity is administered at uniform intervals—as studied originally studied in [4], and further developed by [5]. However, we also show that TSC allows us to go far beyond this case, yielding solutions for arbitrary dosing regimes where the amount of drug administered and the interval between doses can vary arbitrarily. This generalization is important, so long equi-dosing regimens are “a difficult, if not impossible, feat to accomplish in any circumstances, be it a clinical or preclinical study or, even more so, in the real case of a patient taking a drug long term” [6, p. 432].

We also use the multiple-dose dynamics of the Bateman function obtained via TSC to showcase results concerning drug accumulation. For instance, we establish mathematical properties inherent to these dynamics, such as asymptotic periodicity and the convergence to a “Steady State”-a phase where blood concentration dynamics fluctuate within a predetermined range. Additionally, we formulate a new mathematical definition of the “Steady State”, and use it to provide explicit formulas for the asymptotic bounds of the dynamics.

Finally, we showcase the versatility of TSC in extending its application to various established PKPD and PBPK models. Consequently, we formulate multiple-dose dynamics via TSC of bolus injections through intravascular routes and models incorporating finite absorption times, as discussed in [7, 8]. Following the insights of [6], our findings highlight the profound influence of administration routes and the underlying assumptions about drug absorption and elimination on drug accumulation kinetics in plasma.

Our paper is the first use of TSC in PKPD and PBPK applications. This novel application broadens TSC’s scope beyond its established utility in areas such as population dynamics and biology (e.g., [9–12]), economics (e.g., [13–16]), physics (e.g., [17]), machine learning (e.g., [18]), and robotics (e.g., [19]).

The closest work to our proposal is that of [5], which presents exact solutions for equi-dosing regimens within multi-dose pharmacokinetic models incorporating transit compartments. Similarly, the research conducted by [20, 21] provides exact solutions for multiple intermittent intravenous infusions within a single-compartment model. Our research distinguishes itself from these precedents in three fundamental aspects: First, we show that the multi-dose dynamics can be naturally encapsulated within the TSC framework. Specifically, dynamic equations -a generalization of differential and difference equations proposed by TSC—allow us to integrate absorption, elimination, and dose intakes into a single initial value problem. Hence, this approach differs from traditional modeling practices where researchers typically amalgamate multiple conventional ordinary differential equations describing post-dose dynamics. Second, we show that the dynamic equation language to PKPD and PBPK allows a simpler formulation and solution to complex dosing regimens. We demonstrate this by deriving analytical solutions for arbitrary dosing schedules, thus improving upon the equi-dosing assumptions discussed in prior works. Third, our modeling framework is valid for models different from the standard transit compartment systems. An example is the multi-dose dynamics of Physiologically Based Finite Time Pharmacokinetic (PBFTPK) models explored in this paper. With this application, we contribute to the PBFTPK literature by providing multiple-dose dynamics where only single-dose dynamics have been developed (see [7, 8]).

In addition, we provide several new results concerning drug accumulation. First, we offer a new mathematical formulation of the “steady state” for multi-dose dynamics based on TSC. We also provide alternate proofs of several well-established drug accumulation results in medical practice, as described in [6]. Finally, we offer a novel result that asserts that a physician can—theoretically—formulate dosage regimens for blood concentrations to stay within any desired range asymptotically.

The remainder of this paper is organized as follows. Section 2 introduces TSC, derives multiple-dose dynamics stemming from the Bateman Function, and describes its properties. Section 3 provides additional applications of TSC to other PKPD and PBPK models. Section 4 concludes.

## An application of TSC: exploring multi-dose dynamics of the Bateman function

In this section, we employ TSC to deliver analytical solutions for multiple-dose dynamics resulting from the Bateman function under different dosing regimens. The Bateman function is a one-compartment model disposition with first-order absorption and elimination rate, frequently applied in pharmacokinetic analyses of oral dosing. As a solution to an ordinary differential equation initial value problem, we use the Bateman function to exemplify the value of TSC in transitioning from single-dose to multiple-dose dynamics via dynamic equations.

### The Bateman function

The Bateman Function describes the blood concentration dynamics following a single oral dose intake. As shown in [22], the Bateman Function is the solution of a system of linear ordinary differential equations modeling drug absorption and circulation through the gastrointestinal tract. In fact, the Bateman Function is also a solution to a second-order linear differential equation, as shown in (1).

Following [23], the concentration of many orally-administered drugs in plasma over time after a single dose, denoted as *x*(*t*), is well approximated in empirical data by the following “Bateman Function”:

1 $$\begin{aligned} x(t)=\dfrac{\kappa _a F d}{V\,(\kappa _a-\kappa _e)}\; \left( e^{-\kappa _e t}-e^{-\kappa _a t}\right), \end{aligned}$$

where $\kappa _a,\kappa _e$ represent the compound absorption and elimination rate constants, respectively, and *F*, *V*, *d* account for absolute bioavailability, the volume of distribution, and the amount of the oral dose, respectively. It is usually the case that $\kappa _{a}> \kappa _{e} > 0$, meaning the absorption of the drug into the central compartment is faster than the elimination process. However, in some cases $0<\kappa _{a} < \kappa _{e}$, which is known as the flip-flop situation [24]. Although it is possible to deal with the case where both parameters are equal, we will not refer to this case in our theory since it is uncommon to be found in practice.

More broadly, and following Eq. (1), the drug blood concentration dynamics resulting from the administration of a broad spectrum of medications is the solution to the following second-order initial value problem:

2 $$\begin{aligned} {\left\{ \begin{array}{ll} x''(t)+(\kappa _a + \kappa _e) x'(t) + \kappa _e\kappa _a x(t) = 0\\ x'(0) = {\kappa _a \,\gamma \,d }\\ x(0)=0\\ \end{array}\right. }, \end{aligned}$$

where parameter $\gamma$ condenses different combinations of biological parameters depending on the route of administration^1^.

Alternatively, this particular second-order linear equation can be transformed into a system of first-order linear equations by introducing an auxiliary function *y*(*t*). The function $y(t)$ can be biologically interpreted as representing the quantity of the drug present in organs responsible for absorption at a time *t*. This reformulation leads to the following dynamical system

3 $$\begin{aligned} \begin{aligned} {\left\{ \begin{array}{ll} y'(t) = -\kappa _{a} y(t)\\ x'(t) = \kappa _{a}\,\gamma \,y(t) - \kappa _{e} x(t)\\ y(0) = y_{0} = d;\\ x(0) = x_{0} = 0 \end{array}\right. } \end{aligned} \end{aligned}$$

with $$\kappa _{a} > 0$$, $$\kappa _{e} > 0$$, and $$\kappa _a \ne \kappa _e$$.

From a mathematical perspective, we have a system of two autonomous linear first-order differential equations with constant coefficients, for which a closed-form solution is known. For one, the differential equation governing *y*(*t*) represents the process by which the drug amount in the gastrointestinal tract is absorbed, wherein $\kappa _a$ is the absorption rate constant. For another, the dynamics of *x*(*t*) encapsulate the aftermath of the absorption and elimination processes: $\kappa _{a}\gamma y(t)$ denotes the concentration of the drug instantaneously absorbed at time *t* (inflow), while $- \kappa _{e} x(t)$ indicates the drug’s elimination, wherein $\kappa _e$ is the elimination rate constant. The system is coupled with initial conditions $y(0) = y_{0} = d$, and $x(0) = x_{0} = 0$, where *d* represents the quantity of the drug administered orally. This particular choice of initial conditions allows the solution to match equation (1).

Transitioning from single-dose to multi-dose dynamics necessitates a framework capable of accommodating dose intake mechanics beyond what is typically offered by ordinary differential equations. This requirement is met through the development of dynamic equations, which extend traditional differential equations to incorporate discrete events, such as drug intakes, into the model. In the subsequent section, we delineate the foundational elements of this theory, setting the stage for its application to the Bateman function.

### Time scale calculus preliminaries

TSC is an emerging field in mathematics that aims to unify the theory of differential and difference equations by enabling the study of dynamics on more general time domains called time scales. As an introduction, we briefly overview the main definitions of TSC following [25, 26].

#### Basic definitions

As the name suggests, this theory aims to generalize discrete and continuous dynamical systems into more general time sets called time scales. Specifically, a time scale is defined as follows:

##### Definition 1

A *Time Scale* $\mathbb {T}$ is an arbitrary nonempty closed subset of $\mathbb {R}$.

For instance, $\mathbb {R},\; \mathbb {Z},\; \mathbb {N} \cup [0,1]$ are time scales.

##### Definition 2

Let $\mathbb {T}$ be a Time Scale. For $t \in \mathbb {T}$, we define the *forward jump operator* $\sigma : \mathbb {T} \rightarrow \mathbb {T}$ by:

$$\begin{aligned} \sigma (t):= \inf \{s \in \mathbb {T} : s>t\} \end{aligned}$$

Analogously, we define the *backward jump operator*

$$\rho : \mathbb {T} \rightarrow \mathbb {T}$$ by:

$$\begin{aligned} \rho (t):= \sup \{s \in \mathbb {T} : s<t\} \end{aligned}$$

Intuitively, the definition of time scales admits both discrete and continuous sets and sets with both components. As it is expected, dynamics will vary depending on the “structure” of the Time Scale. This leads to the following classification.

##### Definition 3

Let $\mathbb {T}$ a time scale. An element $t \in \mathbb {T}$ is:

1. *right-scattered* if $t<\sigma (t)$.
2. *right-dense* if $t=\sigma (t)$.
3. *left-scattered* if $t>\rho (t)$.
4. *left-dense* if $t=\rho (t)$.
5. *isolated* if $$\rho (t)<t<\sigma (t)$$.
6. *dense* if $$\rho (t)=t=\sigma (t)$$

In other words, the forward and backward operators determine whether, at a certain point, the dynamics resemble a discrete or a continuous scale. An alternative approach is to define another operator called the graininess function:

##### Definition 4

Let $\mathbb {T}$ a time scale. We define the *graininess function* $\mu : \mathbb {T} \longrightarrow \mathbb {R}_{\ge 0}= [0, \infty )$ by:

$$\begin{aligned} \mu (t):= \sigma (t)-t \end{aligned}$$

Observe that if $\mu (t)=0$, then the time scale at *t* is continuous, whereas if $\mu (t)>0$, then the time scale at *t* behaves like a discrete set. Finally, we define a distinguished subset of every time scale that will be useful for other definitions.

##### Definition 5

We define the subset $\mathbb {T}^{\kappa }$ as

$$\begin{aligned} \mathbb {T}^{\kappa }= {\left\{ \begin{array}{ll} \mathbb {T} \setminus (\rho (\sup (\mathbb {T})),\sup (\mathbb {T})] &{} \text {if } \sup (\mathbb {T})<\infty \\ \mathbb {T} &{} \text {if } \sup (\mathbb {T})=\infty \\ \end{array}\right. } \end{aligned}$$

#### Differentiation

We introduce the extended notion of a derivative in an arbitrary time scale.

##### Definition 6

Let $f: \mathbb {T} \rightarrow \mathbb {R}$ be a function and $t \in \mathbb {T}^{\kappa }$. We define $f^{\Delta }(t)$ (provided it exists) to be the number that for every $\epsilon >0$, there exists $\delta >0$ such that there is a neighbourhood $U=(t-\delta ,t+\delta ) \cap \mathbb {T}$ that satisfies:

$$\begin{aligned} |(f(\sigma (t))-f(s))-f^{\Delta }(t)(\sigma (t)-s)| \le \epsilon |\sigma (t)-s|, \quad \forall s \in U \end{aligned}$$

We call $f^{\Delta }(t)$ the *delta-derivative* (or Hilger Derivative) of $f$ at $t$.

The following Theorem shows how this definition extends the notion of the regular derivative and discrete differences:

##### Proposition 1

Assume $f:\mathbb {T} \rightarrow \mathbb {R}$ be a function and let $t \in \mathbb {T}^{\kappa }$. Then the following are valid:

(i) if $f$ is delta-differentiable at $t$, it is continuous at $t$.
(ii) if $f$ is continuous and $t$ is right-scattered, the $f$ is delta-differentiable at $t$ with:
  $$\begin{aligned} f^{\Delta }(t)=\dfrac{f(\sigma (t))-f(t)}{\mu (t)} \end{aligned}$$
(iii) if $t$ is right-dense, then $f$ is delta-differentiable at $t$ if and only if the limit,
  $$\begin{aligned} \lim \limits _{s \rightarrow t} \dfrac{f(t)-f(s)}{t-s} \end{aligned}$$
  exists as a finite number. In this case:
  $$\begin{aligned} f^{\Delta }(t)=\lim \limits _{s \rightarrow t} \dfrac{f(t)-f(s)}{t-s}=f'(t) \end{aligned}$$

In summary, the Hilger derivative behaves like an ordinary derivative in dense domains and like a discrete difference in scattered domains. This enables a single and general notion of “change” in time domains that exhibit both denseness and scatteredness properties.

#### Dynamic equations

Like ordinary differential and difference equations, the TSC allows formulating equations that describe the dynamics of an object in an arbitrary time scale—these are called “dynamic equations”. A dynamic equation of order *p* is a mathematical equation that relates a function and its delta derivatives up to order *p* with respect to a single independent variable *t*. More precisely, it takes the form:

$$\begin{aligned} G(t,f(t),f^{\Delta }(t),\ldots , f^{\Delta ^p}(t))=0 \end{aligned}$$

Additionally, these equations can be combined with initial and boundary conditions to produce specific solutions. The general solution for many dynamic equations is known and thoroughly addressed in [25, 26].

### The equi-dosing regimen

In this section, we apply TSC to model blood concentration levels resulting from multiple successive drug doses under a Bateman function. The key idea behind this proposal is that the specific dynamical system depicted in (3), leading to the Bateman Function, results from assuming dynamics unfold on a quite specific time scale: $\mathbb {R}_{\ge 0} = [0, \infty )$. However, these same dynamics can also potentially describe treatment responses in more general time scales. For instance, consider a simple scenario in which *d* grams of a particular drug is administered every $\tau$ units of time, with the first dose being administered at time $t=0$. Thus, we can visualize the dynamics happening on the following time scale $\mathbb {T}(\tau )$:

4 $$\begin{aligned} \mathbb {T}(\tau )=\bigcup \limits _{n=1}^{\infty }[(n-1)\,\tau , n\, \tau ] = \bigcup \limits _{n=1}^{\infty }I_{n}. \end{aligned}$$

The intervals $I_{n} = [(n-1)\tau , n \tau ]$, for $n=1,2,3,\ldots$, represent the time lapses between each dose administration. The beginning of interval $I_n$ marks the moment the *n*-th dose is administered. In other words, the *n*-th dose is administered at the beginning of interval $I_n$, precisely at time $(n-1)\tau$. Because *d* and $\tau$ are constant, this case is known in the literature as the “Equi-dosing case” [5].

Leveraging TSC tools, we can embed the dynamical system introduced in (3) into a different time scale—$\mathbb {T}(\tau )$. Specifically, this generalization is possible via the Hilger derivative. The exact formulation of this extension is as follows:

5 $$\begin{aligned} \begin{aligned} {\left\{ \begin{array}{ll} y^{\triangle } (t) = -\kappa _{a}y(t)\\ x^{\triangle } (t) = \kappa _{a} \, \gamma \,y(t) -\kappa _{e}x(t) \\ {\textbf {with initial conditions:}} \\ y(0) = d\\ x(0) = 0 \\ {\textbf {and multiplicity conditions:}} \\ \lim \limits _{t \rightarrow n\tau ^+} y(t)=\lim \limits _{t \rightarrow n\tau ^-} y(t)+ \, d; \quad n=1, 2,\ldots , \infty \\ \lim \limits _{t \rightarrow n\tau ^+} x(t)=\lim \limits _{t \rightarrow n\tau ^-} x(t); \quad n=1, 2,\ldots , \infty \end{array}\right. } \end{aligned} \end{aligned}$$

Equation 5 condenses the equi-dosing problem into a single initial value problem using dynamic equations. Note that when considering a single-dose scenario $(\tau \rightarrow \infty )$, the proposed model matches exactly (2) since $\mathbb {T}(\infty )=[0,\infty )=\mathbb {R}_{\ge 0}$, and thus, the Hilger derivative is the same as the ordinary derivative.

In addition to the initial conditions typically used in ordinary differential equations’ initial value problems, multiple-dose dynamics require specifying conditions at dose intake times. Our model assumes that the concentration in the systemic circulation does not rise abruptly when a new dose is administered orally, hence the continuity condition

$$\begin{aligned} \lim \limits _{t \rightarrow n\tau ^+} x(t)=\lim \limits _{t \rightarrow n\tau ^-} x(t) \end{aligned}$$

In terms of the model, this implies that the drug concentration at the time of a new intake is equal to the concentration observed after $\tau$ units of time have elapsed since the last dose administration. Similarly, we require that

$$\begin{aligned} \lim \limits _{t \rightarrow n\tau ^+} y(t)=\lim \limits _{t \rightarrow n\tau ^-} y(t)+ \, d \end{aligned}$$

This condition can be interpreted as follows: when a new drug dose is administered, it immediately enters the gastrointestinal tract, causing an abrupt increase in the amount of medicine to be absorbed by that system by *d* units.

The solution to the above system thus describes the dynamics of *x* and *y* after administering a dose *d* every $\tau$ units of time. Since this model is derived from the “Bateman Function”, the resulting function for *x*(*t*) can be described as its equi-multiple-dose version. The formal result is presented in the following theorem:

#### Theorem 2

[The Equi-multiple-dose Bateman Function]

Consider the Time Scale defined by $\mathbb {T}(\tau )=\bigcup _{n=1}^{\infty } I_{n} = \bigcup _{n=1}^{\infty } [(n-1)\tau , n \tau ]$. Then the initial value problem presented in (5) on Time Scale $\mathbb {T}(\tau )$ has a unique solution given by:

6 $$\begin{aligned} {\left\{ \begin{array}{ll} y(t)= \sum \limits _{n=1}^{\infty } {\mathbb{1}}[t \in I_{n}] \overset{(n)}{y}(t); \quad \overset{(n)}{y}(t) = d \left( \dfrac{1 - \alpha ^{n}}{1 - \alpha } \right) e^{-\kappa _{a}(t - (n-1)\tau )} \\ x(t)= \sum \limits _{n=1}^\infty \mathbb{1}[t \in I_{n}] \overset{(n)}{x}(t); \quad \overset{(n)}{x}(t) = C_{1} e^{-\kappa _{e}(t - (n-1)\tau )} - C_{2} e^{-\kappa _{a}(t - (n-1)\tau )} \\ \text {where} \\ C_{1} = \left( \dfrac{\kappa _{a}\, \gamma \,d}{\kappa _{a} - \kappa _{e}} \right) \left( \dfrac{1 - \beta ^{n}}{1 - \beta } \right) \\ C_{2} = \left( \dfrac{\kappa _{a}\, \gamma \, d}{\kappa _{a} - \kappa _{e}} \right) \left( \dfrac{1 - \alpha ^{n}}{1 - \alpha } \right) \\ \alpha = e^{-\kappa _{a} \tau }, \quad 0< \alpha< 1 \\ \beta = e^{-\kappa _{e} \tau }, \quad 0< \beta < 1 \\ \end{array}\right. } \end{aligned}$$

The function *x*(*t*) is called the *Equi-multiple-dose Bateman Function*.

#### Proof

See Online Appendix B.1.

$$\square$$

Theorem 2 presents an explicit formula for the dynamics of multiple doses, assuming that both the dose amount and the interval between doses remain constant. The solutions are articulated as infinite sums of functions, where $\overset{\scriptstyle {(n)}}{x}(t)$ and $\overset{\scriptstyle {(n)}}{y}(t)$ denote the particular solutions within a cycle, that is, the period between the *n*th and (*n*  + 1)th dose.

### Arbitrary dosing regimens

In this section, we offer a direct extension of the model described in Sect. 2.3 when allowing for dosage regimens with uneven times between doses and possibly different grammages in the doses.

Let $t_n$ be the time the $n-$th dose is administered, and define $\tau _n = t_{n} - t_{n-1}$ be the time elapsed between doses. Let $d_n$ represent the amount of drug administered at time $t_n$. Then, the sequence $\{(\tau _{n},d_n)\}_{n=1}^\infty$ characterizes any conceivable dosage plan.

We must consider the times scale generated by an arbitrary regimen to characterize dynamics in a general setting. Similar to the constant case, this can be formulated as

$$\begin{aligned} \mathbb {T}(\{\tau _n\}_{n=1}^\infty )=\bigcup \limits _{n=1}^\infty I_n(\tau _1,\ldots , \tau _n)=\bigcup \limits _{n=1}^\infty [t_{n-1}, t_{n}] \end{aligned}$$

Notice this is a generalization of $\mathbb {T}(\tau )$, which results from the special case when $\tau _n=\tau$ for all *n*, so that $I_n(\tau _1,\ldots , \tau _n)=I_n=[(n-1)\tau ,n\tau ]$.

As with the equi-dosing plans, the corresponding dynamics are described by a system of dynamic equations. Concretely, for all $t \in \mathbb {T}(\{\tau _n\}_{n=1}^\infty )$, consider the multi-dose dynamics are given by the following system of dynamic equations,

7 $$\begin{aligned} \begin{aligned} {\left\{ \begin{array}{ll} y^{\triangle } (t) = -\kappa _{a}y(t)\\ x^{\triangle } (t) = \gamma \,\kappa _{a} \,y(t) -\kappa _{e}x(t) \\ {\textbf {with initial conditions:}} \\ y(0) = d_{1}\\ x(0) = 0 \\ {\textbf {and multiplicity conditions:}} \\ \lim \limits _{t \rightarrow t_{n-1}^+} y(t) = \lim \limits _{t \rightarrow t_{n-1}^-} y(t) + \, d_{n}; \quad n=2, 3,\ldots , \infty \\ \lim \limits _{t \rightarrow t_{n-1}^+} x(t) = \lim \limits _{t \rightarrow t_{n-1}^-} x(t); \quad n=2, 3,\ldots , \infty \end{array}\right. } \end{aligned} \end{aligned}$$

As in Sect. 2.3, this dynamic system allows for a closed-form solution, which we present in Theorem 3

#### Theorem 3

[The Generalized-multiple-dose Bateman Function]

Provided that $\kappa _{a} \ne \kappa _{e}$ and given any arbitrary dosage schedule given by the sequence $\left\{ (d_{n}, \tau _{n})\right\} _{n=1}^{\infty }$, the solution to the system of dynamic equations presented in (7) is:

8 $$\begin{aligned}y(t)&= \sum \limits _{n=1}^\infty {\mathbb{1}}[t \in I_{n}] \overset{(n)}{y}(t) \\ x(t)&= \sum \limits _{n=1}^\infty {\mathbb{1}}[t \in I_{n}] \overset{(n)}{x}(t) \end{aligned}$$

where

9 $$\begin{aligned} \begin{aligned} {\mathop {y}\limits ^{(n)}}(t)&= \left( \sum \limits _{i=1}^{n-1}\prod \limits _{j=i}^{n-1} d_{i}\alpha _{j} + d_{n} \right) e^{-\kappa _{a}(t - t_{n-1})} \\ {\mathop {x}\limits ^{(n)}}(t)&= C_{1}(n) \, e^{-\kappa _{e}(t - t_{n-1})} - C_{2}(n) \, e^{-\kappa _{a}(t - t_{n-1})} \\ C_{1}(n)&= \dfrac{\kappa _{a}\, \gamma }{\kappa _{a} - \kappa _{e}} \left( \sum \limits _{i=1}^{n-1}\prod \limits _{j=i}^{n-1} d_{i}\alpha _{j} + d_{n} \right) \\ {}&\quad + \dfrac{\kappa _{a}\cdot \gamma }{\kappa _{a} - \kappa _{e}} \left[ \sum \limits _{i=1}^{n-1}\prod \limits _{j=i}^{n-1} d_{i}\beta _{j} - \sum \limits _{i=1}^{n-1}\prod \limits _{j=i}^{n-1} d_{i}\alpha _{j} \right] \\ C_{2}(n)&= \dfrac{\kappa _{a}\, \gamma \ }{\kappa _{a} - \kappa _{e}} \left( \sum \limits _{i=1}^{n-1}\prod \limits _{j=i}^{n-1} d_{i}\alpha _{j} + d_{n} \right) \\ \alpha _{s}&= e^{-\kappa _{a}\tau _{s}},\, \beta _{s} = e^{-\kappa _{e}\tau _{s}}, \, s=1,2,3,\ldots , n\ge 1.\\ \end{aligned} \end{aligned}$$

The function *x*(*t*) is called the *Generalized-multiple-dose Bateman Function*.

#### Proof

See Online Appendix B.2. $\square$

**Fig. 1:**
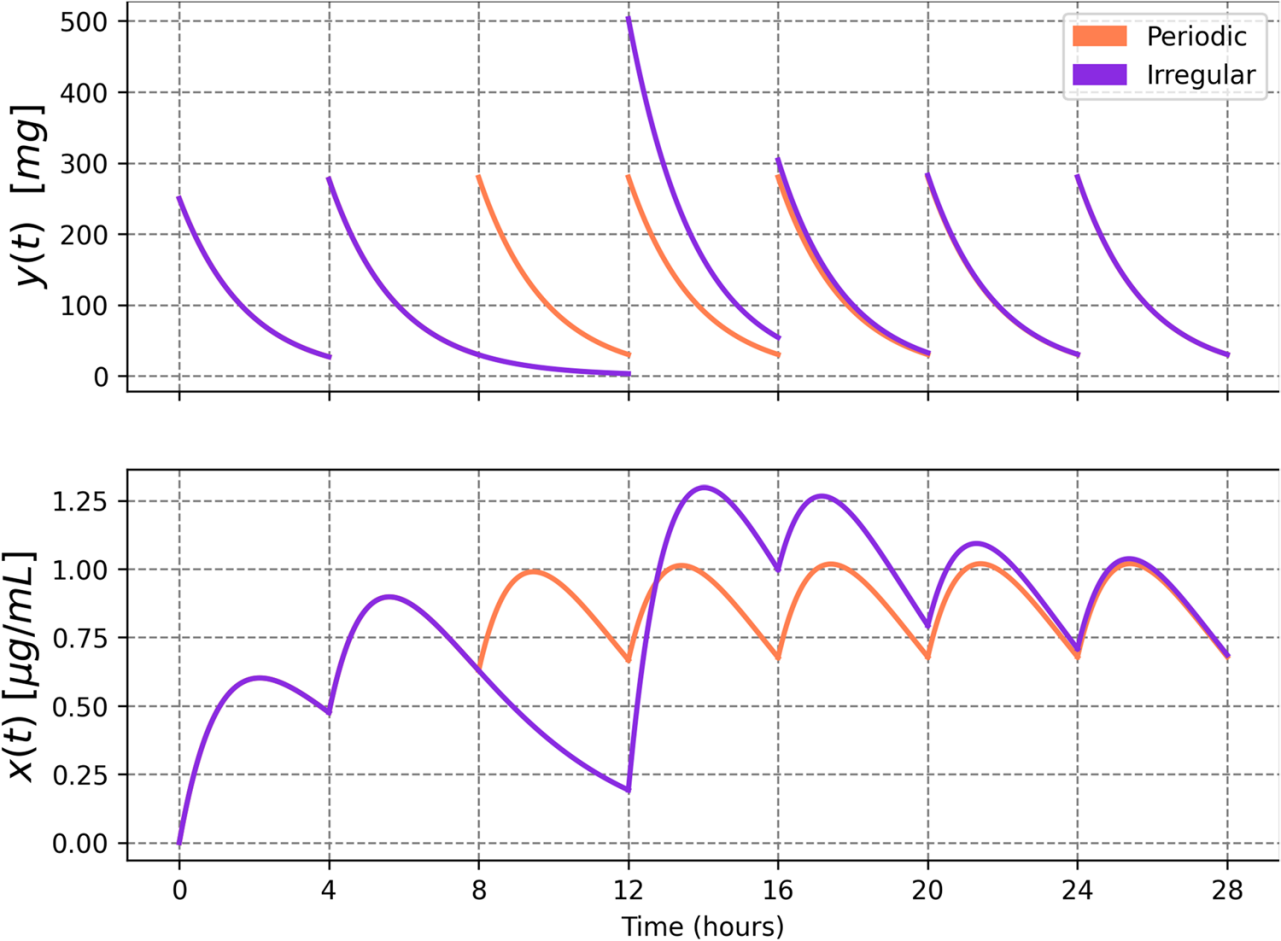
Periodic dose vs. irregular dose—illustration. The top panel shows the amount of the drug in the intestinal tract, and the lower panel depicts the drug concentration in the bloodstream

As illustrated in Fig. 1, irregular dosing schedules can lead to dynamics that diverge significantly from the “periodic” solutions outlined in the previously discussed section. The blue line charts the blood concentration resulting from a consistent regimen of administering 250 mg of a hypothetical drug every 4 h. Conversely, the orange line depicts a scenario where the patient skips the third dose and subsequently takes a double dose (500 mg) at the next scheduled time. While blood concentration levels eventually converge to a similar dynamic, this example underscores how deviations in dosing times and amounts can markedly affect the dynamics in the short term and may lead to prolonged variations if such patterns are consistently followed.

### Properties of the multiple-dose Bateman functions

In this section, we exhibit properties of the Equi-multiple-dose Bateman Function. Similar properties can be established for the Generalized-multiple-dose Bateman Function.

#### Standard pharmacokinetic/pharmacodynamic quantities:

We begin by calculating several typical pharmacokinetic/pharmacodynamic variables, such as the Area Under the Curve (AUC), maximum plasma concentration and the peak time in each cycle. Propositions 4 through 7 summarize these results.

##### Proposition 4

(Area under the curve—$\text {AUC}_{I_{n}}$) The area under the concentration–time curve in the interval $I_{n} = [(n-1)\tau , n\,\tau ]$, denoted as $\text {AUC}_{I_{n}}$, satisfies the following expression:

10 $$\begin{aligned} \text {AUC}_{I_{n}} = \dfrac{\kappa _{a}\, d\, \gamma }{\kappa _{a} - \kappa _{e}} \left[ \dfrac{1}{\kappa _{e}} \left( 1 - \beta ^{n} \right) - \dfrac{1}{\kappa _{a}} \left( 1 - \alpha ^{n} \right) \right] \end{aligned}$$

##### Proof

See Online Appendix B.3. $\square$

##### Proposition 5

Let $\text {AUC}_{[0,\infty ]}$ be the area under the concentration–time curve in the case of a single dose between $t=0$ and $t\rightarrow \infty$. The $\text {AUC}_{[0,\infty ]}$ satisfies the following expression:

11 $$\begin{aligned} \text {AUC}_{[0, \infty )} = \dfrac{\kappa _{a}\, d\, \gamma }{\kappa _{a} - \kappa _{e}} \left[ \dfrac{1}{\kappa _{e}} - \dfrac{1}{\kappa _{a}} \right] \end{aligned}$$

##### Proof

See Online Appendix B.4. $\square$

##### Proposition 6

The time at which the plasma concentration reaches its maximum value in period $I_{n} = [(n-1)\tau , n\,\tau ]$ is denoted as ${\mathop {t}\limits ^{(n)}}_{\text {max}}$ and satisfies the equation:

12 $$\begin{aligned} {\mathop {t}\limits ^{(n)}}_{\text {max}} = (n-1)\tau + \dfrac{1}{\kappa _{a} - \kappa _{e}} \ln \left( \dfrac{\kappa _{a} C_{2}}{\kappa _{e} C_{1}} \right) \end{aligned}$$

which is always a positive number regardless of whether $\alpha > \beta$ or $\beta > \alpha$. Here, $C_1$ and $C_{2}$ are the constants defined in Theorem 2.

##### Proof

See Online Appendix B.5. $\square$

##### Proposition 7

The maximum plasma concentration in the period $I_{n} = [(n-1)\tau , n\tau ]$, denoted as ${\mathop {x}\limits ^{(n)}}_{\text {max}}$, satisfies the following formula:

13 $$\begin{aligned} {\mathop {x}\limits ^{(n)}}_{\text {max}} = {\mathop {x}\limits ^{(n)}}\left( {\mathop {t}\limits ^{(n)}}_{\text {max}}\right) = C_{1} \left( \frac{\kappa _{a}C_{2}}{\kappa _{e}C_{1}} \right) ^{-\dfrac{\kappa _{e}}{\kappa _a - \kappa _e}} - C_{2} \left( \frac{\kappa _{a}C_{2}}{\kappa _{e}C_{1}} \right) ^{-\dfrac{\kappa _{a}}{\kappa _a - \kappa _e}} \end{aligned}$$

where $C_{1}$ and $C_{2}$ are defined in Theorem 2.

**Fig. 2:**
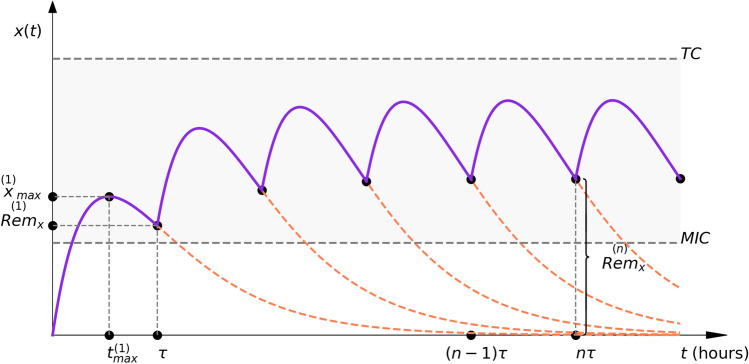
The equi-multiple-dose Bateman function—illustration

To summarize the previous results, we have graphically illustrated them in Fig. 2.

#### Asymptotic behavior of the solutions

In this section, we investigate the plasma concentration after numerous dose administrations by observing the behavior of the sequence of functions $\{{\mathop {x}\limits ^{(n)}}\}_{n =0}^\infty$ as $n \rightarrow \infty$. While these results pertain to the equi-dose regimen case, some can be extended to arbitrary dosage regimens in several situations.

##### Theorem 8

[Asymptotic periodicity]

For a fixed choice of parameters ($\kappa _a,\kappa _e,\gamma$), $\kappa _a \ne \kappa _e$, the sequence of functions ${\mathop {x}\limits ^{(0)}}, {\mathop {x}\limits ^{(1)}}, \ldots ,$ is asymptotically $\tau$-periodic, meaning

$$\begin{aligned} \lim \limits _{n \rightarrow \infty } \left[ \sup \limits _{t \in I_n} \; \Big |{\mathop {x}\limits ^{(n)}}(t)-{\mathop {x}\limits ^{(n-1)}}(t-\tau ) \Big | \right] =0 \end{aligned}$$

##### Proof

See Online Appendix B.6. $\square$

Theorem 8 asserts that the dynamics of plasma concentration stabilize into identical cycles after multiple doses have been administered. The predictability of concentration dynamics after several dose intakes suggests that solutions reach a “steady state”, where the blood concentration dynamics exhibit a regular pattern. A similar result can be established for many arbitrary dose regimens in Online Appendix A. Consequently, we formalize the notion of a steady state as follows:

##### Definition 7

[Steady state] Let $\epsilon >0$ and let

$$\begin{aligned} N_\epsilon := \min \Bigg \{ n \in \mathbb {N} \cup \{0\} : \forall m \ge n, \sup \limits _{t \in I_{m}} \; \Big |x^{(m)}(t)-x^{(m-1)}(t-\tau ) \Big |<\epsilon \Bigg \} \end{aligned}$$

We define the $\epsilon$-*steady-state* of the model as the time scale

$$\begin{aligned} \mathbb {T}_\epsilon ^{ss}(\tau ) := \bigcup \limits _{n \ge N_\epsilon } I_n \subseteq \mathbb {T}(\tau ) \end{aligned}$$

Furthermore, we denote by

$$\begin{aligned} \overset{(s.s.)}{x_{\epsilon }} := x \Big \vert _{\mathbb {T}^{ss}_{\epsilon }(\tau )} \end{aligned}$$

as the $\epsilon$-*steady-state* dynamics.

An immediate corollary of Theorem 8 is that “steady states” always exist for any conceivable dosage plan. More precisely, we know that a an $\epsilon$-steady-state always exist for any $\epsilon >0$, and for any set of parameters $\kappa _a,\kappa _e,\gamma$, provided that $\kappa _a \ne \kappa _e$. Furthermore, this definition justifies studying the limiting behavior of standard pharmacokinetic/pharmacodynamic quantities by considering their limits when $n \rightarrow \infty$.

Additionally, asymptotic periodicity implies that it is possible to study the long-term behavior of patients following a specific dosage plan. In particular, it allows for examining the range of possible concentrations a patient may exhibit after being administered a particular drug dosage for a long period.

##### Definition 8

[Therapeutic Range] Let $\epsilon >0$ and let

$$\begin{aligned} TR_\epsilon ^{\text {s.s.}}=\text {Range}(\overset{(s.s.)}{x_\epsilon }) \end{aligned}$$

be the range of values a solution can exhibit when it has achieved its $\epsilon -$steady state. We define the therapeutic range or safety range as:

14 $$\begin{aligned} \text {TR}^{\text {s.s.}} = [ \underline{\text {SS}}, \overline{\text {SS}} ]= \bigcap \limits _{\epsilon>0} TR_\epsilon ^{\text {s.s.}}= \bigcap \limits _{\epsilon >0} \text {Range}(\overset{(s.s.)}{x_\epsilon }) \end{aligned}$$

In brief, the therapeutic or safety range is the set of possible concentrations an individual can exhibit when $t \rightarrow \infty$. We now find explicit formulas for $\underline{\text {SS}}$ and for $\overline{\text {SS}}$. To achieve this, consider the following sequences indexed by *n*.

##### Definition 9

[Remainder] We will refer to the plasma concentration of medication that remains in the circulatory system after the *n*-th period, $I_{n} = [(n-1)\tau , n \tau ]$, as that period’s remainder. We denote this quantity by ${\mathop {{\textbf {Rem}}_{x}}\limits ^{(n)}}$. Formally:

15 $$\begin{aligned} {\mathop {{\textbf {Rem}}_{x}}\limits ^{(n)}} := {\mathop {x}\limits ^{(n)}}(n\,\tau ) = \dfrac{\kappa _{a}\,d\,\gamma }{\kappa _{a} - \kappa _{e}} \left[ \left( \dfrac{1 - \beta ^{n}}{1 -\beta } \right) \beta - \left( \dfrac{1 - \alpha ^{n}}{1 -\alpha } \right) \alpha \right] \end{aligned}$$

Since both $0< \alpha = e^{-\kappa _{a}\tau } < 1$ and $0< \alpha = e^{-\kappa _{a}\tau } < 1$ as $n \rightarrow \infty$, the remainder converges to a quantity ${\mathop {{\textbf {Rem}}_{x}}\limits ^{(\infty )}}$. Moreover, by definition, this quantity constitutes the lower bound of the therapeutic range. Hence:

16 $$\begin{aligned} \underline{\text {SS}}= & {} {\mathop {{\textbf {Rem}}_{x}}\limits ^{(\infty )}} := \lim \limits _{n\rightarrow \infty } {\mathop {{\textbf {Rem}}_{x}}\limits ^{(n)}}\nonumber \\ {}= & {} \dfrac{\kappa _{a}\,d\,\gamma }{\kappa _{a} - \kappa _{e}} \left[ \left( \dfrac{\beta }{1 -\beta } \right) - \left( \dfrac{\alpha }{1 -\alpha } \right) \right] \end{aligned}$$

Furthermore, it can be verified that ${\mathop {{\textbf {Rem}}_{x}}\limits ^{(n)}}$ is always a positive number for each *n*, regardless of whether $\alpha > \beta$ or $\beta > \alpha$. Likewise, it follows that $\underline{\text {SS}}={\mathop {{\textbf {Rem}}_{x}}\limits ^{(\infty )}} > 0$.

The following proposition carries out a similar procedure to find the upper bound of the therapeutic range

##### Proposition 9

The maximum plasma concentration in the steady state converges to a positive number and is given by the following expression:

17 $$\begin{aligned} \overline{\text {SS}}= & {} \lim \limits _{n \rightarrow \infty } {\mathop {x}\limits ^{(n)}}_{\text {max}} = \frac{\kappa _{a}d\gamma }{\kappa _{a} - \kappa _{e}} \nonumber \\ {}{} & {} \times \Bigg [ \frac{1}{1 - \beta } \Bigg ( \frac{\kappa _{a}(1-\beta )}{\kappa _{e}(1-\alpha )} \Bigg )^{-\frac{\kappa _{e}}{\kappa _{a} - \kappa _{e}}} \nonumber \\ {}{} & {} \qquad - \frac{1}{1 - \alpha } \Bigg (\frac{\kappa _{a}(1-\beta )}{\kappa _{e}(1-\alpha )} \Bigg )^{-\frac{\kappa _{a}}{\kappa _{a} - \kappa _{e}}} \Bigg ] \end{aligned}$$

##### Proof

See Online Appendix B.7. $\square$

Critically, plasma concentration asymptotic behavior depends on the dosage schedule $(d,\tau )$. Theorem 10 demonstrates how different schedules result in different therapeutic ranges:

##### Theorem 10

[Steady-state bounds] For a given vector physiological parameters, $(\kappa _{a}, \kappa _{e}, \gamma )$, let $\overline{SS}(d,\tau )$ and $\underline{SS}(d,\tau )$ be the upper and lower bounds of the therapeutic range in the steady-state seen as functions of $(d,\tau )$.

- If the personalized dose *d* is increased while maintaining the personalized dose regimen $\tau$ constant for a patient, then $\underline{SS} (d,\tau )$ increases and $\overline{SS} (d,\tau )$ increases.
- If the time between doses $\tau$ is increased while keeping the dose *d* constant, then $\underline{SS} (d,\tau )$ decreases with a limit of zero and $\overline{SS} (d,\tau )$ decreases up to a positive limit.

##### Proof

See Online Appendix B.8. $\square$

Moreover, we can show that the Therapeutic Range is well-defined in that $\overline{SS} > \underline{SS}$, as well as characterize how different dosage plans alter its width. Theorem 11 exhibits these results

##### Theorem 11

[Width of the Therapeutic Range] The maximum and minimum plasma concentrations, $\overline{SS}$ and $\underline{SS}$, in the steady-state always satisfy the following inequality:

18 $$\begin{aligned} \overline{SS}(d,\tau ) > \underline{SS}(d,\tau ) \end{aligned}$$

The width of the therapeutic range, defined as $\ell (d,\tau ) := \overline{SS}(d,\tau ) - \underline{SS}(d,\tau )$, increases with respect to the dose *d* and the dosage regime $\tau$. Moreover, if the dose is kept constant ($d>0$), then,

19 $$\begin{aligned} \lim \limits _{\tau \rightarrow \infty } \ell (d,\tau ) = \dfrac{\kappa _{a}\,d\, \gamma }{\kappa _{a} - \kappa _{e}} \left[ \left( \dfrac{\kappa _{a}}{\kappa _{e}} \right) ^{-\frac{\kappa _{e}}{\kappa _{a} - \kappa _{e}}} - \left( \dfrac{\kappa _{a}}{\kappa _{e}} \right) ^{-\frac{\kappa _{a}}{\kappa _{a} - \kappa _{e}}} \right] >0 \end{aligned}$$

##### Proof

See Online Appendix B.9. $\square$

Lastly, it is possible to characterize the area under the curve for the limiting cycle. This is given by

$$\begin{aligned} \text {AUC}^{s.s.}= \lim \limits _{n \rightarrow \infty } \text {AUC}_{I_{n}} \end{aligned}$$

This quantity holds an interesting relationship with the single-dose AUC, as documented by [6] and as formally proved in the following result.

##### Theorem 12

[Equality of areas under the curves] Let $\text {AUC}_{I_{n}}^{\,\,s.s.}$ be the area under the concentration–time curve in the period $I_{n} = [(n-1)\tau , n\tau ]$. Then,

20 $$\begin{aligned} \text {AUC}_{[0,\infty )} = \text {AUC}^{\,\,s.s.} \end{aligned}$$

##### Proof

See Online Appendix B.10. $\square$

The area under the concentration–time curve (AUC) is a critical measure quantifying medication absorption into the systemic circulation. Theorem 12 establishes that the AUC for a single dose is equivalent to the AUC for each cycle in a steady state. In other words, when prescribing an evenly spaced dosage plan with a fixed grammage, a medical practitioner can expect the AUC in the steady state, which is frequently unknown, to be equivalent to that of the single-dose scenario, which is frequently determined through clinical studies.

**Fig. 3:**
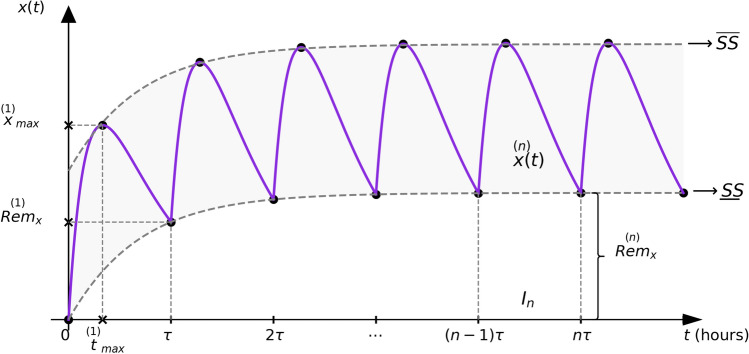
Asymptotic behavior of the equi-multiple-dose Bateman function

To summarize the previous results concerning the asymptotic behavior of the Generalized Function, we have graphically illustrated them in Fig. 3.

#### Long-term treatments.

Many long-term treatments require patients to adhere to a strict dosage regimen for several years, if not their entire lives. Some cases in question involve patients prescribed drugs for high blood pressure or HIV and non-disease treatments such as contraceptive pills. To facilitate the process for the patient, medical practitioners usually prescribe fixed-grammage doses to be taken at evenly spaced intervals. Furthermore, it is well known through practice that drug concentration in the blood stabilizes when prescribing these kinds of dosage schedules—a result we have now mathematically established to be always true in Sect. 2.5.2. Thus, the long-term behavior of these particular treatments can be well described using the asymptotic theory of the Equi-multiple-dose Bateman functions.

In general, the objective of many of these treatments is to maintain concentration levels within a desired range $[\underline{R},\bar{R}]$, where $\bar{R}>\underline{R}>0$ [27, 28]). The lower bound is the Minimum Inhibitory Concentration (MIC), which represents the concentration above which the drug is effective. The upper bound is the Toxic Concentration (TC), which denotes concentrations above which the drug could potentially harm the user. As a result, an effective dosage plan maintains drug concentration levels after several doses within this range. In terms of the model, this means that

$$\begin{aligned} \text {MIC}= \underline{R} \le \underline{\text {SS}} < \overline{\text {SS}} \le \bar{R}= \text {TC} \end{aligned}$$

From this description of the medical problem, two questions naturally arise: for a given set of parameters $(\kappa _a,\kappa _e,\gamma )$ characterizing a patient’s kinetics, (i) does there always exist a dosage plan $(d,\tau )$ that results in a successful treatment for any $[\underline{R},\bar{R}]$? and, (ii) provided there is a solution, how should a dosage plan be designed to be effective?

The answer to these questions is possible by studying the Equi-multiple-dose Bateman function’s asymptotics. Specifically, we have established that the asymptotic behavior of the multi-dose dynamics is a function of $(d,\tau )$, meaning that the set of effective dosage schedules can be described as

$$\begin{aligned} \mathcal {E}(\underline{R},\bar{R};\kappa _a,\kappa _e,\gamma )= \Big \{(d,\tau ) \in \mathbb {R}_{>0} \times \mathbb {R}_{>0} : \underline{\text {SS}}(d,\tau ;\kappa _a,\kappa _e,\gamma ) \ge \underline{R}, \;\; \overline{\text {SS}}(d,\tau ;\kappa _a,\kappa _e,\gamma ) \le \bar{R} \Big \} \end{aligned}$$

##### Theorem 13

For any $\bar{R}>\underline{R}>0$ and physiological parameters $(\kappa _a,\kappa _e,\gamma )$ with $\kappa _a \ne \kappa _e$, the set $\mathcal {E}(\underline{R},\bar{R};\kappa _a,\kappa _e,\gamma )$ is never empty.

##### Proof

See Online Appendix B.11. $\square$

Theorem 13 importance is twofold. From a medical standpoint, it assures us that there always exists a dosage plan-specifically, one featuring constant intervals between intakes and a fixed dosage-that ensures effective treatment for each patient. From a mathematical perspective, it guarantees that methods searching for these solutions can always find effective dosage plans for any patient and any imposed medical requirement.

In our particular framework, one could (theoretically) rely on our knowledge of the asymptotic behavior of the Equi-multiple-dose Bateman Equation to find effective dose schedules. For example, a health practitioner can fix desired long-run concentration levels $[\underline{\text {SS}}^*,\overline{\text {SS}}^*] \subseteq [\underline{R},\bar{R}]$ for their patient. Assuming the biological parameters $(\kappa _a,\kappa _e,\gamma )$ were known, and effective dosage $(\tau ^*,d^*)$ could be theoretically found by solving the non-linear system of equations given by

$$\begin{aligned} \begin{aligned} \underline{\text {SS}}^*&=\underline{SS}(\tau ^*,d^*,\kappa _a,\kappa _e,\gamma )\\ \overline{\text {SS}}^*&=\overline{SS}(\tau ^*,d^*,\kappa _a,\kappa _e,\gamma ) \end{aligned} \end{aligned}$$

Unfortunately, given that such an approach is contingent upon the choice of a particular model and its unknown parameters, it does not offer a universally reliable tool in practice. However, the proof of Theorem 13 does theoretically justify recurrent medical practices. For instance, it implies that the solutions to this problem can be (locally) expressed as functions of the parameters, namely:

$$\begin{aligned} \begin{aligned} \tau ^*&=\tau ^*(\underline{\text {SS}}^*,\overline{\text {SS}}^*,\kappa _a,\kappa _e,\gamma )\\ d^*&=d^*(\underline{\text {SS}}^*,\overline{\text {SS}}^*,\kappa _a,\kappa _e,\gamma ) \end{aligned} \end{aligned}$$

In other words, the dependence of the solutions on the biological parameters endorses the well-known fact that not every drug dosage plan can be effective for every person.

## Other applications

This section showcases additional applications of Time Scale Calculus (TSC) to other PKPD and PBPK models. In particular, we explore multiple-dose dynamics of bolus injections through intravascular routes and models incorporating finite absorption times.

### Additional application 1: bolus injections via intravascular routes

We consider a scenario where a drug is administered as a multidose bolus dose via an intravascular route. Let *x*(*t*) denote the plasma concentration of the drug at time *t*, $t_n$ be the time the *n*th dose is administered, $\tau _n = t_{n} - t_{n-1}$ be the time elapsed between doses, and $\delta _{n}$ denote the concentration of the drug delivered intravenously for dose *n*. In this context, the sequence $\{(\tau _{n},\delta _n)\}_{n=1}^\infty$ outlines any potential dosing regimen. Therefore, the Time Scale suitable for this analysis is defined as:

$$\begin{aligned} \mathbb {T}(\{\tau _n\}_{n=1}^\infty )=\bigcup \limits _{n=1}^\infty I_n(\tau _1,\ldots , \tau _n)=\bigcup \limits _{n=1}^\infty I_n=\bigcup \limits _{n=1}^\infty [t_{n-1}, t_{n}] \end{aligned}$$

Given that the model only incorporates a central compartment to describe elimination dynamics, the dynamics of the model can be articulated through dynamic equations as follows:

21 $$\begin{aligned} {\left\{ \begin{array}{ll} x^{\triangle }(t) = -\kappa _{e}x(t) \\ {\textbf {with initial condition:}} \\ x(0) = \delta _{1}\\ {\textbf {and multiplicity conditions:}} \\ \lim \limits _{t \rightarrow t_{n-1}^+} x(t) = \lim \limits _{t \rightarrow t_{n-1}^-} x(t) + \delta _{n}; \quad n = 2, 3, \ldots , \infty \end{array}\right. } \end{aligned}$$

where $\kappa _{e}$ is the elimination rate constant.

The analytical solution to the initial value problem formulated in (21) is given in the following Theorem:

#### Theorem 14

[The Generalized-multiple-dose bolus injection function]

Given any arbitrary dosage schedule given by the sequence $\left\{ (\delta _{n}, \tau _{n})\right\} _{n=1}^{\infty }$, the solution to the system of dynamic equations presented in (21) is:

22 $$\begin{aligned} x(t)&= \sum \limits _{n=1}^{\infty } \mathbb{1}[t \in I_{n}] \overset{\scriptstyle {(n)}}{x}(t); \quad \overset{\scriptstyle {(n)}}{x}(t) = \left( \sum \limits _{i=1}^{n-1} \prod \limits _{j=i}^{n-1}\delta _{i}\beta _{j} + \delta _{n} \right) e^{-\kappa _{e}(t - t_{n-1})}, \quad \beta _{s} = e^{-\kappa _{e}\tau _{s}} \end{aligned}$$

#### Proof

See Online Appendix C.1. $\square$

**Fig. 4:**
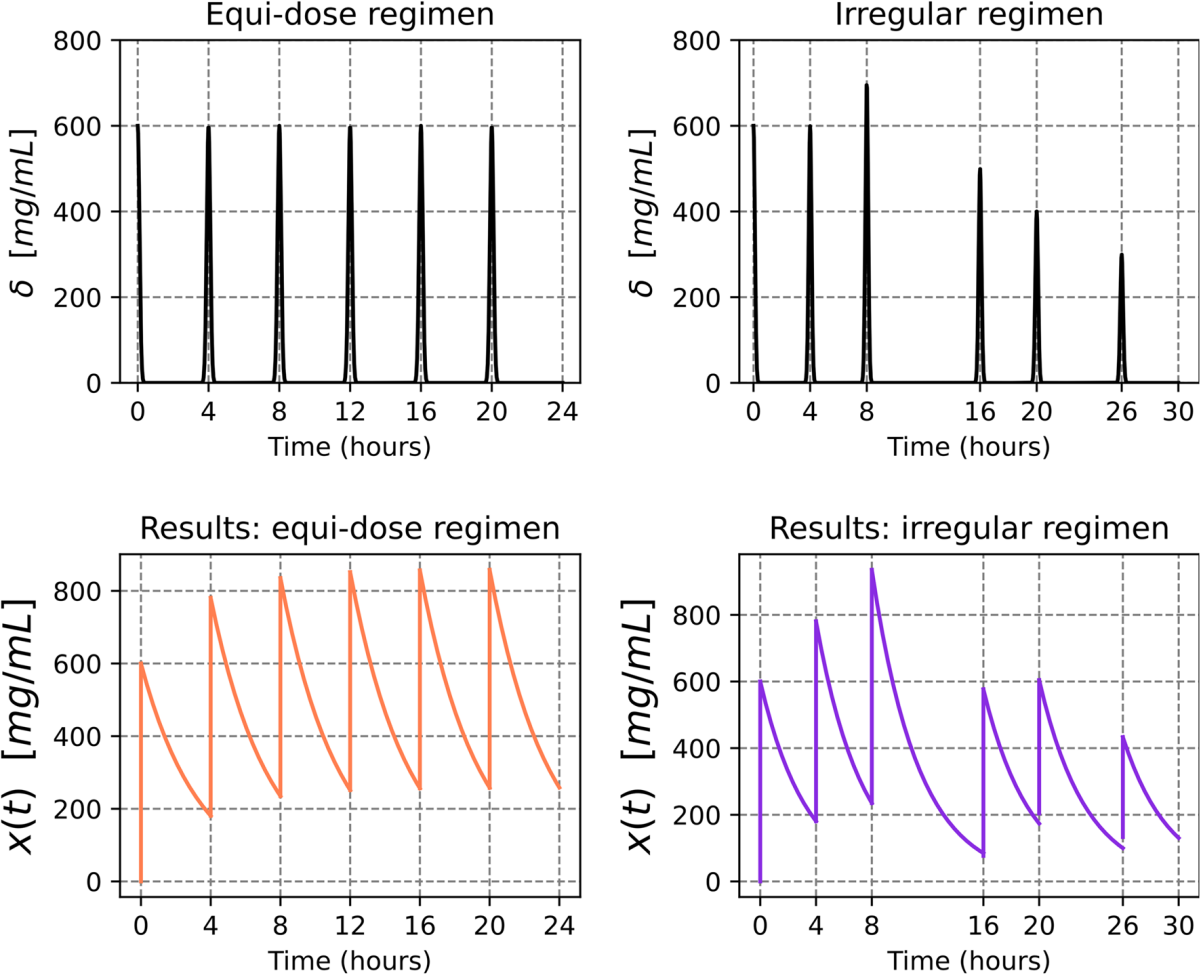
Multiple intravenous bolus dosing—illustration. The simulation shows an equi-dose regimen (left-hand side figures) with $d = 600$ mg/mL and $\tau = 6$ h, and an irregular regimen (right-hand side figures) with $d_{n} = [600, 600, 700, 500, 400, 300]$ mg/mL and $\tau _{n} = [4, 4, 8, 4, 6, 4]$ h. In the simulation, $\kappa _{e}\,=\,0.3838$ h$^{-1}$

Figure 4 uses Theorem 14 to demonstrate the dynamics of multiple doses under two distinct dosing regimens. The left panel displays the dynamics of traditional equi-dosing, while the right panel depicts an irregular dosing regimen. This comparison underscores how plasma concentration profiles can significantly differ due to variations in dosing schedules. As suggested by the functional form retrieved in Theorem 14, this is a consequence of the dependency he dependency of the concentration during cycle *n*, $\overset{\scriptstyle {(n)}}{x}(t)$, on the cumulative history of preceding doses $\left( \overset{\scriptstyle {(n)}}{x}(t)=\overset{\scriptstyle {(n)}}{x}(t;\tau _{n-1},\tau _{n-2},\ldots ,\tau _1)\right)$.

### Application 2: multple-dose physiologically-based finite time pharmacokinetics models

Despite being a well-established model, the Bateman function has faced criticism, such as that articulated by [29], for its reliance on assumptions that may not accurately reflect physiological realities. One significant flaw identified by the authors is the presumption of an infinite absorption time for each dose administration. This does not align with the characteristics of some drugs known for their slow and incomplete absorption profiles, like those belonging to classes II,III, or IV.

Physiologically-based finite Time Pharmacokinetics (PBFTPK) models constitute a response to this critique, introducing the concept of a Finite Absorption Time (F.A.T). Essentially, the F.A.T. theory postulates two different phases following a dose intake. First, drug concentration dynamics are determined jointly by absorption and elimination forces, which can be referred to as the assimilation phase. However, due to biological constraints, this phase is finite, and drug kinetics enter a second phase termed the clearance phase. In this stage, the drug concentration is governed solely by the elimination dynamics because absorption is no longer possible (e.g., the drug is no longer present in the gastrointestinal tract or beyond the respective absorptive sites).

Seminal works like [7, 8] have focused on single-dose PBFTPK models. We now extend these ideas to multiple dosing via TSC. Consider a regimen where a drug is administered in doses of $d_n$ milligrams every $\tau _n$ hours, with absorption occurring only within the first $s_n$ hours, where $s_n \le t_n$. As in other models, the sequence $\{(\tau _{n},\delta _n)\}_{n=1}^\infty$ defines any conceivable dosing plan. However, the time scale here diverges from that in prior models, particularly in accommodating the intervals between doses as $I_{n} = [t_{n-1}, s_{n}] \cup [s_{n}, t_{n}]$, with $[t_{n-1}, s_{n}]$ representing the assimilation phase and $[s_{n}, t_{n}]$ the clearance phase. Thus, the new time scale incorporates both F.A.T. and dosing intervals, expressed as:

$$\begin{aligned} \mathbb {T}(\{s_n\}_{n=1}^\infty ,\{\tau _n\}_{n=1}^\infty )=\bigcup \limits _{n=1}^\infty I_n=\bigcup \limits _{n=1}^\infty \left( [t_{n-1}, s_{n}] \cup [s_{n}, t_{n}]\right) \end{aligned}$$

Within this framework, while maintaining the one-compartment model of Bateman with first-order absorption and elimination rates, the equations for multi-dose dynamics can be expressed as:

23 $$\begin{aligned} \begin{aligned} {\left\{ \begin{array}{ll} y^{\triangle } (t) = -\kappa _{a}y(t)\\ x^{\triangle } (t) =\kappa _{a} \, \gamma \,y(t) -\kappa _{e}x(t) \\ {\textbf {with initial conditions:}} \\ \overset{(1)}{y}(0) = d_{1} \\ \overset{(1)}{x}(0) = 0 \\ {\textbf {and multiplicity conditions:}} \\ y(t_{n-1}) = d_{n}; \quad n=1,2,\ldots ,\infty \\ \lim \limits _{t \rightarrow t_{n-1}^+} x(t) = \lim \limits _{t \rightarrow t_{n-1}^-} x(t); \quad n=2, 3,\ldots ,\infty \\ y(s_{n}) = 0; \quad n=1,2,\ldots ,\infty \\ \lim \limits _{t \rightarrow s_{n}^+} x(t) = \lim \limits _{t \rightarrow s_{n}^-} x(t); \quad n=1,2,\ldots ,\infty \end{array}\right. } \end{aligned} \end{aligned}$$

Equation (23) contains the initial value problem for the multiple-dose PBFTPK model. As in Sect. 2.3, we impose multiplicity assumptions at dose intake times, namely, the first and second on the list. However, introducing F.A.T. requires the imposition of two additional multiplicity conditions. The third, and most critical, condition is expressed as:

$$\begin{aligned} y(s_{n}) = 0; \quad n=1,\ldots ,\infty , \end{aligned}$$

which implies that the absorption drug is depleted after time $s_n$. Indeed, given that $y^{\triangle } (t) = -\kappa _{a}y(t)=0$, it follows that $y(t)=0$ for $t \in [s_{n}, t_{n}]$, thus effectively encapsulating the theoretical dynamics of finite absorption.

The fourth condition:

$$\begin{aligned} \lim \limits _{t \rightarrow s_{n}^+} x(t) = \lim \limits _{t \rightarrow s_{n}^-} x(t); \quad n=1,\ldots ,\infty \end{aligned}$$

ensures the continuity of blood concentration dynamics even after the drug has been fully absorbed.

The solution to this dynamic equation is detailed in Theorem 15:

#### Theorem 15

[Multiple-dose PBFTPK drug concentration]

Given any arbitrary dosage schedule given by the sequence $\left\{ (\delta _{n}, \tau _{n})\right\} _{n=1}^{\infty }$, and a sequence of F.A.T. $\{s_n\}_{n=1}^\infty$, the solution to the system of dynamic equations presented in (23) is:

24 $$\begin{aligned} {\left\{\begin{array}{ll}y(t)&= \sum \limits _{n=1}^{\infty } {\mathbb{1}}[t \in I_{n}^{1}] \,\,\overset{\scriptstyle {(n)}}{y_{1}}(t), \quad \overset{\scriptstyle {(n)}}{y_{1}}(t) = d_{n} e^{-\kappa _{a}(t - t_{n-1})} \\ x(t) &= \sum \limits _{n=1}^\infty {\mathbb{1}}[t \in I_{n}^{1}] \,\,\overset{\scriptstyle {(n)}}{x_{1}}(t)+\mathbb{1}[t \in I_{n}^{2}] \,\,\overset{\scriptstyle {(n)}}{x_{2}}(t); \quad {\left\{ \begin{array}{ll} \overset{\scriptstyle {(n)}}{x_{1}}(t) = C_{1}(n) e^{-\kappa _{e}(t - t_{n-1})} - C_{2}(n) e^{-\kappa _{a}(t - t_{n-1})} \\ \overset{\scriptstyle {(n)}}{x_{2}}(t) = C_{3}(n) e^{-\kappa _{e}(t - s_{n})} \end{array}\right. } \\ \text {where} \\ I_{n}^{1} &= [t_{n-1}, s_{n}],\textit{(Assimilation Phase)}; \quad I_{n}^{2} = [s_{n}, t_{n}],\textit{(Clearance Phase)}; \quad n = 1,\ldots ,\infty \\ C_{1}(n) &= \frac{\kappa _{a}\cdot \gamma }{\kappa _{a} - \kappa _{e}} d_{n} +\frac{\beta }{B}\left[ \dfrac{\kappa _{a}\cdot \gamma }{\kappa _{a} - \kappa _{e}} (B - A) \left( \sum \limits _{i = 1}^{n-1} \beta ^{n-1-i}\, d_{i}\right) \right] \\ C_{2}(n) &= \dfrac{\kappa _{a}\cdot \gamma }{\kappa _{a} - \kappa _{e}} d_{n} \\ C_{3}(n) &{}= \dfrac{\kappa _{a}\cdot \gamma }{ \kappa _{a} - \kappa _{e}} (B - A) \left[ \sum \limits _{i = 1}^{n-1} \beta ^{n-1-i}\, d_{i} \right] \\ \alpha &= e^{-\kappa _{a} \tau }, \quad 0< \alpha< 1 \quad \beta = e^{-\kappa _{e} \tau }, \quad 0< \beta< 1 \\ A &= e^{-\kappa _{a} \sigma }, \quad 0< A< 1 \quad B = e^{-\kappa _{e} \sigma }, \quad 0< B < 1 \\ \alpha ^{n} &= e^{-\kappa _{a}t_{n}}, \quad \beta ^{n} = e^{-\kappa _{e}t_{n}}, \quad \alpha ^{n-1} A = e^{-\kappa _{a}s_{n}}, \quad \beta ^{n-1} B = e^{-\kappa _{e}s_{n}}; \quad n = 1,2,\ldots ,\infty \\ \alpha ^{0} &= \beta ^{0} = 1, \; t_0=0.\end{array}\right. } \end{aligned}$$

#### Proof

See Online Appendix C.2.

$$\square$$

**Fig. 5:**
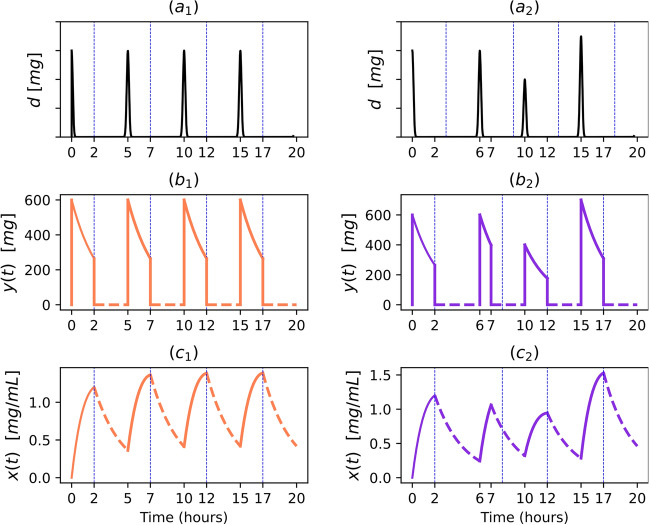
Multiple-dose F.A.T dynamics—illustration. The simulation shows an equi-dose regimen (left-hand side figures) with $d = 600$ mg, $\tau = 5$ h, $s_n = 2$ h and an irregular regimen (right-hand side figures) with $d_{n} = [600, 600, 400, 700]$ mg and $\tau _{n} = [6, 4, 5, 5]$ h, and $s_n = 2$ h. In the simulation, $\kappa _{a} = 0.42$ h$^{-1}$ $\kappa _{e} = 0.4$ h$^{-1}$, $\gamma = 0.00449$ mL$^{-1}$. Vertical dotted lines indicate the Finite Absorption times

Figure 5 showcases the dynamics of multiple doses incorporating Finite Absorption Time (F.A.T). The left panel depicts an equi-dosing scenario, where both the administered dose and the interval between dosing and the F.A.T remain constant. Conversely, the right panel introduces variations in these parameters across doses. A notable feature introduced by incorporating F.A.T is a distinctive kink in the blood concentration dynamics due to the solution provided in Theorem 24 being a piecewise function.^2^ This change corresponds to the phase shift, in which blood concentration dynamics transit from being determined by both absorption and elimination forces, to being governed only by elimination.

## Conclusions

This paper applies TSC to explore the dynamics of multiple drug doses, emphasizing how TSC’s tools simplify our formulation of these complex dynamics. TSC offers a comprehensive framework ideally suited to the dual nature of drug administration, which encompasses the continuous processes of absorption, metabolism, and elimination, as well as the discrete instances of drug intake. Through applying TSC, we develop the multi-dose dynamics for several key pharmacokinetic (PK) models and establish analytical expressions for the trajectory of blood concentrations. Specifically, we formulate multiple-dose dynamics of several flagship PK models using TSC and derive new analytical formulas of the blood concentration dynamics. A critical advantage of TSC is that it enables the formulation of entire multi-dose dynamics as a simple, unique initial value problem. This simplification in the language allows us to handle intricate dosing patterns, like those featuring irregular dose timings and quantities, and to accommodate non-conventional absorption-elimination dynamics, such as those with a finite absorption period.

Although our discussion has been limited to a few simple examples that facilitate the exposition of the technique, TSC constitutes an ideal toolkit to formulate and solve more complex PKPD and PBPK models. For instance, by using the same techniques shown in this paper, it is possible to find analytical solutions for higher-order transit compartment models under unrestricted dosing regimens. Likewise, TSC offers possibilities to model coupled dynamical systems that incorporate continuous processes and discrete events. For example, it enables the simultaneous modeling of multi-dose drug administration aimed at bacterial eradication alongside models of bacterial population dynamics. Furthermore, TSC opens avenues for exploring optimization strategies for multiple-dose prescriptions via dynamic programming in time scales, a field well developed in recent years.

## Supplementary Information

Below is the link to the electronic supplementary material.

Supplementary file 1 (pdf 373 KB)

## Data Availability

No datasets were generated or analysed during the current study.

## Footnotes

1 For example, [23] indicates that for an oral administration (P.O.) of the drug $\gamma =F/V$ [ml$^{-1}$] where *F* is the bioavailability and *V* is the volume of distribution, while for an intravenous administration (I.V.) of the drug, $\gamma$ involves the rate constants governing formation, $\kappa _{f}$, and elimination, $\kappa _{m}$, as well as systemic availability, $F_{H}(m)$, which is the ratio of the amount of metabolite leaving the liver to the amount of metabolite formed, and distribution volume $V_{m}$ for the metabolite.

2 These characteristics appear in other works exploring blood concentration dynamics after single or multiple successive zero-order input rates under F.A.T schemes (see [30]).
