# Supplementary material for: Time Scale Calculus: a new approach to multi-dose pharmacokinetic modeling
Source: J Pharmacokinet Pharmacodyn. 2024 Jul 25;51(6):825–39. doi: 10.1007/s10928-024-09920-z (PMC11579191; doi:10.1007/s10928-024-09920-z)
Supplement: Supplementary file 1 — (pdf 373 KB) [file 10928_2024_9920_MOESM1_ESM.pdf]

# Appendix for:

# Time Scale Calculus: A New Approach to Multi-Dose Pharmacokinetic Modeling.

## Contents


**A Appendix - Asymptotic periodicity for arbitrary dose schedules. 1**

**B Appendix - Omitted proofs - Bateman function 4**

B.1 Proof of Theorem 2. . . . . . 4
B.2 Proof of Theorem 3. . . . . . 6
B.3 Proof of Proposition 4 . . . . . . 9
B.4 Proof of Proposition 5 . . . . . . 9
B.5 Proof of Proposition 6 . . . . . . 10
B.6 Proof of Theorem 8 . . . . . . 10
B.7 Proof of Proposition 9 . . . . . . 10
B.8 Proof of Theorem 10. . . . . . 11
B.9 Proof of Theorem 11 . . . . . . 12
B.10 Proof of Proposition 12 . . . . . . 12
B.11 Proof of Theorem 13 . . . . . . 12

**C Appendix - Omitted proofs - Other applications 17**

C.1 Proof of Theorem 14 . . . . . . 17
C.2 Proof of Theorem 15 . . . . . . 18

# A Appendix - Asymptotic periodicity for arbitrary dose schedules.

Similarly to the equi-dose model, we can affirm the existence of asymptotic periodicity for non-equi-dose regimes, thereby establishing the concept of a steady state. Specifically, we can extend Theorem 8 through the following theorem.

**Theorem 16** (Asymptotic periodicity)**.** *Consider an arbitrary dosage schedule* $\{(d_n, \tau_n)\}_{n=1}^{\infty}$ *such that* $\lim_{n\to\infty} d_n = d$ *and* $\lim_{n\to\infty} \tau_n = \tau$*, for some* $d > 0$ *and* $\tau > 0$*.*

*For a fixed choice of parameters* $(\kappa_a, \kappa_e, \gamma)$*,* $\kappa_a \neq \kappa_e$*, the sequence of functions* $\overset{(0)}{x}, \overset{(1)}{x}, \cdots$*, resulting from the Generalized Bateman function with schedule* $\{(d_n, \tau_n)\}_{n=1}^{\infty}$ *is asymptotically* $\tau-$*periodic, meaning*

$$\lim_{n\to\infty}\left[\sup_{t\in I_n}\left|\overset{(n)}{x}(t) - \overset{(n-1)}{x}(t-\tau)\right|\right] = 0$$

*Proof.* We begin this proof with a preliminary proposition:

**Proposition 17.** *Consider the remainder sequences* $\overset{(n)}{\boldsymbol{Rem}_x}$ *and* $\overset{(n)}{\boldsymbol{Rem}_y}$ *presented in Theorem 18. Then*

$$\limsup_{n\to\infty} \overset{(n)}{\boldsymbol{Rem}_x} = \overline{\boldsymbol{Rem}}_x < \infty \qquad \textit{and} \qquad \limsup_{n\to\infty} \overset{(n)}{\boldsymbol{Rem}_y} = \overline{\boldsymbol{Rem}}_y < \infty$$

*Proof.* Since $\{\tau_n\}_{n=1}^{\infty}$ and $\{d_n\}_{n=1}^{\infty}$ are convergent sequences, they are also bounded. Let $\bar{\tau} = \max_n \tau_n$ and $\bar{d} = \max_n d_n$.

To prove the assertion, it suffices to show that both sequences are bounded under the given hypothesis. First, notice that for all $n$, $\alpha_n = e^{-\kappa_a \tau_n}$ is bounded. Let $\bar{\alpha}$ be this bound, with $\bar{\alpha} \in (0, 1)$. This assertion is also true for $\beta_n = e^{-\kappa_e \tau_n}$, whose bound we will denote $\bar{\beta} \in (0, 1)$.

It follows that

$$\overset{(n)}{\mathbf{Rem}_y} = \sum_{i=1}^{n}\prod_{j=i}^{n} d_i\alpha_j \leq \bar{d}\sum_{i=1}^{n}(\bar{\alpha})^{n-i} = \bar{d}\sum_{i=0}^{n-1}(\bar{\alpha})^{i} \leq \bar{d}\sum_{i=0}^{\infty}(\bar{\alpha})^{i} = \frac{\bar{d}}{1-\bar{\alpha}} < \infty$$

given the geometric series is convergent insofar as $\bar{\alpha} \in (0, 1)$.

Similarly,

$$\begin{aligned}
\overset{(n)}{\mathbf{Rem}_x} &= \frac{\kappa_a \cdot \gamma}{\kappa_a - \kappa_e}\left[\sum_{i=1}^{n}\prod_{j=i}^{n} d_i\beta_j - \sum_{i=1}^{n}\prod_{j=i}^{n} d_i\alpha_j\right] \\
&\leq \frac{\kappa_a \cdot \gamma}{\kappa_a - \kappa_e}\left[\sum_{i=1}^{n}\prod_{j=i}^{n} d_i\beta_j + \sum_{i=1}^{n}\prod_{j=i}^{n} d_i\alpha_j\right] \\
&\leq \frac{\kappa_a \cdot \gamma\bar{d}}{\kappa_a - \kappa_e}\left[\sum_{i=1}^{n}(\bar{\beta})^{n-i} + \sum_{i=1}^{n}(\bar{\alpha})^{n-i}\right] \\
&= \frac{\kappa_a \cdot \gamma\bar{d}}{\kappa_a - \kappa_e}\left[\sum_{i=0}^{n-1}(\bar{\beta})^{i} + \sum_{i=0}^{n-1}(\bar{\alpha})^{i}\right] \\
&\leq \frac{\kappa_a \cdot \gamma\bar{d}}{\kappa_a - \kappa_e}\left[\sum_{i=0}^{\infty}(\bar{\beta})^{i} + \sum_{i=0}^{\infty}(\bar{\alpha})^{i}\right]
\end{aligned}$$

$$\leq \frac{\kappa_a \cdot \gamma \bar{d}}{\kappa_a - \kappa_e}\left[\frac{1}{1-\bar{\beta}} + \frac{1}{1-\bar{\alpha}}\right] < \infty$$

■

Returning to the original proof, since $t_{n-1} = \tau_{n-1} + t_{n-2}$, it follows that

$$\begin{aligned}
\left|\overset{(n)}{x}(t) - \overset{(n-1)}{x}(t-\tau)\right| &= \Bigg| \left[\left(\frac{\kappa_a \cdot \gamma \cdot (\overset{(n-1)}{\mathbf{Rem}_y} + d_n)}{\kappa_a - \kappa_e} + \overset{(n-1)}{\mathbf{Rem}_x}\right) e^{-\kappa_e (t - t_{n-1})} - \left(\frac{\kappa_a \cdot \gamma \cdot (\overset{(n-1)}{\mathbf{Rem}_y} + d_n)}{\kappa_a - \kappa_e}\right) e^{-\kappa_a (t - t_{n-1})}\right] \\
&- \left[\left(\frac{\kappa_a \cdot \gamma \cdot (\overset{(n-2)}{\mathbf{Rem}_y} + d_{n-1})}{\kappa_a - \kappa_e} + \overset{(n-2)}{\mathbf{Rem}_x}\right) e^{-\kappa_e (t - \tau - t_{n-2})} - \left(\frac{\kappa_a \cdot \gamma \cdot (\overset{(n-2)}{\mathbf{Rem}_y} + d_n)}{\kappa_a - \kappa_e}\right) e^{-\kappa_a (t - \tau - t_{n-2})}\right] \Bigg| \\
&= |A_n(t)\, e^{-\kappa_e t_{n-2}} - B_n(t)\, e^{-\kappa_a t_{n-2}}| \\
&\leq |A_n(t)| + |B_n(t)|
\end{aligned}$$

where

$$\begin{aligned}
|A_n(t)| &= \left|\left(\frac{\kappa_a \cdot \gamma \cdot (\overset{(n-1)}{\mathbf{Rem}_y} + d_n)}{\kappa_a - \kappa_e} + \overset{(n-1)}{\mathbf{Rem}_x}\right) e^{-\kappa_e (t - \tau_{n-1})} - \left(\frac{\kappa_a \cdot \gamma \cdot (\overset{(n-2)}{\mathbf{Rem}_y} + d_{n-1})}{\kappa_a - \kappa_e} + \overset{(n-2)}{\mathbf{Rem}_x}\right) e^{-\kappa_e (t - \tau)}\right| \\
&\leq \left|\left(\frac{\kappa_a \cdot \gamma \cdot (\overset{(n-1)}{\mathbf{Rem}_y} + d_n)}{\kappa_a - \kappa_e} + \overset{(n-1)}{\mathbf{Rem}_x}\right) - \left(\frac{\kappa_a \cdot \gamma \cdot (\overset{(n-2)}{\mathbf{Rem}_y} + d_{n-1})}{\kappa_a - \kappa_e} + \overset{(n-2)}{\mathbf{Rem}_x}\right)\right| + \left|e^{-\kappa_e (t - \tau_{n-1})} - e^{-\kappa_e (t - \tau)}\right| \\
&= \left|\left(\frac{\kappa_a \cdot \gamma \cdot (\overset{(n-1)}{\mathbf{Rem}_y} + d_n)}{\kappa_a - \kappa_e} + \overset{(n-1)}{\mathbf{Rem}_x}\right) - \left(\frac{\kappa_a \cdot \gamma \cdot (\overset{(n-2)}{\mathbf{Rem}_y} + d_{n-1})}{\kappa_a - \kappa_e} + \overset{(n-2)}{\mathbf{Rem}_x}\right)\right| + \left|e^{\kappa_e \tau_{n-1}} - e^{\kappa_e \tau}\right| e^{-\kappa_e t}
\end{aligned}$$

and

$$\begin{aligned}
|B_n(t)| &= \left|\left(\frac{\kappa_a \cdot \gamma \cdot (\overset{(n-1)}{\mathbf{Rem}_y} + d_n)}{\kappa_a - \kappa_e}\right) e^{-\kappa_a (t - \tau_{n-1})} - \left(\frac{\kappa_a \cdot \gamma \cdot (\overset{(n-1)}{\mathbf{Rem}_y} + d_n)}{\kappa_a - \kappa_e}\right) e^{-\kappa_a (t - \tau)}\right| \\
&\leq \left|\left(\frac{\kappa_a \cdot \gamma \cdot (\overset{(n-1)}{\mathbf{Rem}_y} + d_n)}{\kappa_a - \kappa_e}\right) - \left(\frac{\kappa_a \cdot \gamma \cdot (\overset{(n-1)}{\mathbf{Rem}_y} + d_n)}{\kappa_a - \kappa_e}\right)\right| + \left|e^{-\kappa_a (t - \tau_{n-1})} - e^{-\kappa_a (t - \tau)}\right| \\
&= \left|\left(\frac{\kappa_a \cdot \gamma \cdot (\overset{(n-1)}{\mathbf{Rem}_y} + d_n)}{\kappa_a - \kappa_e}\right) - \left(\frac{\kappa_a \cdot \gamma \cdot (\overset{(n-1)}{\mathbf{Rem}_y} + d_n)}{\kappa_a - \kappa_e}\right)\right| + \left|e^{\kappa_e \tau_{n-1}} - e^{\kappa_e \tau}\right| e^{-\kappa_e t}
\end{aligned}$$

So that

$$\sup_{t \in I_n} |A_n(t)| \leq \left|\left(\frac{\kappa_a \cdot \gamma \cdot (\overset{(n-1)}{\mathbf{Rem}_y} + d_n)}{\kappa_a - \kappa_e} + \overset{(n-1)}{\mathbf{Rem}_x}\right) - \left(\frac{\kappa_a \cdot \gamma \cdot (\overset{(n-2)}{\mathbf{Rem}_y} + d_{n-1})}{\kappa_a - \kappa_e} + \overset{(n-2)}{\mathbf{Rem}_x}\right)\right| + |e^{\kappa_e \tau_{n-1}} - e^{\kappa_e \tau}|$$

$$\sup_{t \in I_n} |B_n(t)| \leq \left|\left(\frac{\kappa_a \cdot \gamma \cdot (\overset{(n-1)}{\mathbf{Rem}_y} + d_n)}{\kappa_a - \kappa_e}\right) - \left(\frac{\kappa_a \cdot \gamma \cdot (\overset{(n-1)}{\mathbf{Rem}_y} + d_n)}{\kappa_a - \kappa_e}\right)\right| + |e^{\kappa_e \tau_{n-1}} - e^{\kappa_e \tau}|$$

and, consequently

$$\sup_{t \in I_n} \left|\overset{(n)}{x}(t) - \overset{(n-1)}{x}(t-\tau)\right| \leq \sup_{t \in I_n} |A_n(t)| + \sup_{t \in I_n} |B_n(t)| = C_n$$

Now note that

$$\limsup_{n\to\infty} \sup_{t\in I_n} |A_n(t)| \leq \left| \left( \frac{\kappa_a \cdot \gamma(\overline{\mathbf{Rem}}_y + d)}{\kappa_a - \kappa_e} + \overline{\mathbf{Rem}}_x \right) - \left( \frac{\kappa_a \cdot \gamma(\overline{\mathbf{Rem}}_y + d)}{\kappa_a - \kappa_e} + \overline{\mathbf{Rem}}_x \right) \right| + |e^{\kappa_e \tau} - e^{\kappa_e \tau}| = 0$$

$$\limsup_{n\to\infty} \sup_{t\in I_n} |B_n(t)| \leq \left| \left( \frac{\kappa_a \cdot \gamma \cdot (\overline{\mathbf{Rem}}_y + d)}{\kappa_a - \kappa_e} \right) - \left( \frac{\kappa_a \cdot \gamma \cdot \overline{\mathbf{Rem}}_y + d)}{\kappa_a - \kappa_e} \right) \right| + |e^{\kappa_e \tau} - e^{\kappa_e \tau}| = 0$$

Hence

$$\limsup_{n\to\infty} C_n = 0$$

Invoking the squeeze theorem, it follows that

$$\lim_{n\to\infty} \left[ \sup_{t\in I_n} \left| \overset{(n)}{x}(t) - \overset{(n-1)}{x}(t-\tau) \right| \right] = 0$$

∎

# B Appendix - Omitted proofs - Bateman function

## B.1 Proof of Theorem 2.

*Proof.* We will use mathematical induction.

- $n = 1$. In this case, the Hilger derivative inside interval $I_1 = [0, \tau]$ coincides with the usual derivative. Hence, the solution of the first equation of the system (5) under the first initial condition is,

$$\frac{dy}{dt} = -\kappa_a y \implies y(t) = d\, e^{-\kappa_a t} \tag{25}$$

  Using this solution we can find the solution of the second equation of the system (5) under the second initial condition is,

$$\begin{aligned} \frac{dx}{dt} &= \kappa_a\, \gamma\, y(t) - \kappa_e x(t) \\ \implies x'(t) + \kappa_e\, x(t) &= \kappa_a\, \gamma\, d\, e^{-\kappa_a t} \end{aligned} \tag{26}$$

  This is a non-homogeneous first-order linear differential equation with constant coefficients. Its integrating factor is $\mu = e^{\kappa_e t}$. Multiplying for the integrating factor and integrating with respect to $t$ we obtain,

$$e^{\kappa_e t}\, x(t) = \kappa_a\, \gamma\, d\, \frac{e^{-(\kappa_a - \kappa_e)t}}{-(\kappa_a - \kappa_e)} + C \tag{27}$$

  Now using the initial conditions: $y(0) = \gamma\, d$, $x(0) = 0$ we can find the constant of integration,

$$C = \frac{\kappa_a\, \gamma\, d}{\kappa_a - \kappa_e} \tag{28}$$

  So, the solution of the second equation is,

$$x(t) = \frac{\kappa_a\, \gamma\, d}{\kappa_a - \kappa_e} \left(e^{-\kappa_e t} - e^{-\kappa_a t}\right) \quad t \in [0, \tau] \tag{29}$$

- $n > 1$. We proceed by mathematical induction.

  **Induction hypothesis.**

  For $t \in I_n = [(n-1)\tau, n\,\tau]$ and $n > 1$ equations (6) are satisfied.

  **Induction thesis (what must be proved)**
  For $t \in I_{n+1} = [n\tau, (n+1)\,\tau]$ the initial value problem,

$$\begin{cases} \overset{(n+1)\triangle}{y}(t) = -\kappa_a \overset{(n+1)}{y}(t) \\ \overset{(n+1)\triangle}{x}(t) = \kappa_a\, \gamma \overset{(n+1)}{y}(t) - \kappa_e \overset{(n+1)}{x}(t) \\ \text{with I.C.} \\ \overset{(n+1)}{y}(n\tau) = \overset{(n)}{y}(n\tau) + d \\ \overset{(n+1)}{x}(n\tau) = \overset{(n)}{x}(n\tau) \end{cases} \tag{30}$$

  has explicit solution:

$$
\begin{cases}
\overset{(n+1)}{y}(t) = \gamma\, d \left(\dfrac{1-\alpha^{n+1}}{1-\alpha}\right) e^{-\kappa_a (t-n\tau)} \\
\overset{(n)}{x}(t) = \tilde{C}_1 e^{-\kappa_e (t-n\tau)} - \tilde{C}_2 e^{-\kappa_a (t-n\tau)} \\
\text{where} \\
\tilde{C}_1 = \left(\dfrac{\kappa_a\, \gamma\, d}{\kappa_a - \kappa_e}\right)\left(\dfrac{1-\beta^{n+1}}{1-\beta}\right) \\
\tilde{C}_2 = \left(\dfrac{\kappa_a\, \gamma\, d}{\kappa_a - \kappa_e}\right)\left(\dfrac{1-\alpha^{n+1}}{1-\alpha}\right)
\end{cases} \tag{31}
$$

To prove the solution for $\overset{(n+1)}{y}(t)$ holds, we will start by finding an equivalent expression for the first initial condition. For the induction hypothesis, we have,

$$
\begin{aligned}
\overset{(n+1)}{y}(n\tau) &= \overset{(n)}{y}(n\tau) + \gamma\, d \\
&= \gamma\, d \left(\frac{1-\alpha^n}{1-\alpha}\right) e^{-\kappa_a \tau} + \gamma\, d \\
&= \gamma\, d \left(\frac{1-\alpha^n}{1-\alpha}\right) \alpha + \gamma\, d \\
&= \gamma\, d \left[\left(\frac{1-\alpha^n}{1-\alpha}\right) \alpha + 1\right] \\
\overset{(n+1)}{y}(n\tau) &= \gamma\, d \left(\frac{1-\alpha^{n+1}}{1-\alpha}\right)
\end{aligned} \tag{32}
$$

Since the Hilger derivative in the interior of the interval $I_{n+1}$ is the derivative in the usual sense we solve the initial value problem,

$$
\begin{aligned}
&\frac{d}{dt}\overset{(n+1)}{y}(t) = -\kappa_a \overset{(n+1)}{y}(t) \\
\Longrightarrow\ &\overset{(n+1)}{y}(t) = \overset{(n+1)}{y}(n\tau) e^{-\kappa_a (t-n\tau)} \\
&\overset{(n+1)}{y}(t) = \gamma\, d \left(\frac{1-\alpha^{n+1}}{1-\alpha}\right) e^{-\kappa_a (t-n\tau)}
\end{aligned} \tag{33}
$$

Now, we will prove that the solution for $\overset{(n+1)}{x}(t)$ is satisfied in the interval $I_{n+1}$. Using the induction hypothesis again and also taking into account the structure of the differential equation which is: non-homogeneous, first-order linear with constant coefficients and the non-homogeneous part is a multiple of the previously found solution for $\overset{(n+1)}{y}(t)$ (exponential form), we can suppose that the solution has the form

$$
\overset{(n+1)}{x}(t) = \tilde{C}_1 e^{-\kappa_e (t-n\tau)} - \tilde{C}_2 e^{-\kappa_a (t-n\tau)} \tag{34}
$$

where we only have to find the value of the constants, $\tilde{C}_1, \tilde{C}_1$. For this we will use the initial conditions of the problem and the induction hypothesis.

$$
\begin{aligned}
&\overset{(n+1)}{x}(n\tau) = \overset{(n)}{x}(n\tau) \\
\Longrightarrow\ &\tilde{C}_1 - \tilde{C}_2 = C_1 e^{-\kappa_e \tau} - C_2 e^{-\kappa_a \tau} \\
&\tilde{C}_1 - \tilde{C}_2 = C_1 \beta - C_2 \alpha
\end{aligned} \tag{35}
$$

On the other hand, deriving $\overset{(n+1)}{x}(t)$ with respect to $t$ and evaluating in $n\tau$ we obtain a second equation for the constants we want to find.

$$\begin{aligned}
\frac{d}{dt}\overset{(n+1)}{x}(t)\Big|_{t=n\tau} &= -\kappa_e\tilde{C}_1 + \kappa_a\tilde{C}_2 = \kappa_a\overset{(n+1)}{y}(n\tau) - \kappa_e\overset{(n+1)}{x}(n\tau)\\
-\kappa_e\tilde{C}_1 + \kappa_a\tilde{C}_2 &= \kappa_a\overset{(n+1)}{y}(n\tau) - \kappa_e\overset{(n)}{x}(n\tau)\\
-\kappa_e\tilde{C}_1 + \kappa_a\tilde{C}_2 &= \kappa_a\left(\frac{1-\alpha^{n+1}}{1-\alpha}\right) - \kappa_e\overset{(n)}{x}(n\tau)\\
-\kappa_e\tilde{C}_1 + \kappa_a\tilde{C}_2 &= \kappa_a\left(\frac{1-\alpha^{n+1}}{1-\alpha}\right) - \kappa_e\left(C_1\beta - C_2\alpha\right)
\end{aligned} \tag{36}$$

Now, multiplying the first equation, (35), on both sides by $\kappa_e$ and adding term by term with the second equation, (36), and simplifying we have,

$$\tilde{C}_2 = \frac{\kappa_a\gamma\, d}{\kappa_a-\kappa_e}\left(\frac{1-\alpha^{n+1}}{1-\alpha}\right) \tag{37}$$

Finally, substituting this result in the first equation (35) and simplifying we obtain,

$$\tilde{C}_1 = \frac{\kappa_a\gamma\, d}{\kappa_a-\kappa_e}\left(\frac{1-\beta^{n+1}}{1-\beta}\right) \tag{38}$$

■

## B.2 Proof of Theorem 3.

*Proof.* We prove this theorem via two separate propositions.

**Proposition 18** (Recursive Formula for the Generalized Bateman Function)**.** *Provided that $\kappa_a \neq \kappa_e$ then given any arbitrary dosage schedule given by the sequence $\{(d_n, \tau_n)\}_{n=1}^{\infty}$, the solution to the system of dynamic equations presented in (7) is:*

$$\begin{aligned}
y(t) &= \sum_{n=1}^{\infty}\mathbb{1}[t\in I_n]\overset{(n)}{y}(t)\\
x(t) &= \sum_{n=1}^{\infty}\mathbb{1}[t\in I_n]\overset{(n)}{x}(t)
\end{aligned} \tag{39}$$

*where*

$$\begin{aligned}
\overset{(n)}{y}(t) &= \left(\overset{(n-1)}{\mathbf{Rem}_y} + d_n\right)e^{-\kappa_a(t-t_{n-1})}, \qquad t\in I_n = [t_{n-1}, t_n]\\
\overset{(n)}{x}(t) &= C_1(n)\, e^{-\kappa_e(t-t_{n-1})} - C_2(n)\, e^{-\kappa_a(t-t_{n-1})}, \qquad t\in I_n = [t_{n-1}, t_n]\\
C_1(n) &= \frac{\kappa_a\,\gamma}{\kappa_a-\kappa_e}\left(\overset{(n-1)}{\mathbf{Rem}_y} + d_n\right) + \overset{(n-1)}{\mathbf{Rem}_x}\\
C_2(n) &= \frac{\kappa_a\,\gamma}{\kappa_a-\kappa_e}\left(\overset{(n-1)}{\mathbf{Rem}_y} + d_n\right)
\end{aligned} \tag{40}$$

$$\begin{aligned}
\textit{where}\ \ \overset{(n)}{\mathbf{Rem}_y} = \overset{(n)}{y}(t_n) &\quad \textit{is the remainder for } y(t) \textit{ at the end of interval } I_n,\\
\textit{and}\ \ \overset{(n)}{\mathbf{Rem}_x} = \overset{(n)}{x}(t_n) &\quad \textit{is the remainder for } x(t) \textit{ at the end of interval } I_n
\end{aligned}$$

*Proof.* To prove these recurrence formulas, we will solve the initial value problem (IVP) (7) in a generic $n$-th interval, $I_n = [t_{n-1}, t_n]$, taking into account the initial and multiplicity conditions.

Denote as $\overset{(n)}{\mathbf{Rem}_x}$ the value of the drug concentration in the blood plasma at the end of the interval, $I_n$, that is,

$$\overset{(n)}{\mathbf{Rem}_x} = \overset{(n)}{x}(t_n) \tag{41}$$

where $\overset{(n)}{x}(t)$ is the solution of the IVP with $t \in I_n$. Because this quantity is the blood concentration in the body just before the next drug administration, we call it the remainder for $x(t)$ in the $n-th$ interval. Likewise, we denote as $\overset{(n)}{\mathbf{Rem}_y}$ is the amount of drug left in the intestinal tract of the interval at the end of $I_n$, that is,

$$\overset{(n)}{\mathbf{Rem}_y} = \lim_{t \to t_n^-} \overset{(n)}{y}(t) \tag{42}$$

where $\overset{(n)}{y}(t)$ is the solution of IVP with $t \in I_n$. We call it the remainder for $y(t)$ in the $n-th$ interval.

- The first equation of the system (7) is independent of $x(t)$. Therefore its solution is:

$$\begin{aligned} &\frac{d\overset{(n)}{y}}{dt} = -\kappa_a \overset{(n)}{y} \Longrightarrow \overset{(n)}{y}(t) = Ce^{-\kappa_a t} \\ &\text{where} \quad C = \overset{(n)}{y}(t_{n-1}^{+})e^{\kappa_a t_{n-1}} \qquad \text{(initial condition)} \\ &\Longrightarrow \overset{(n)}{y}(t) = \overset{(n)}{y}[t_{n-1}^{+}]e^{-\kappa_a(t-t_{n-1}}; \qquad t \in I_n \\ &\therefore \quad \overset{(n)}{y}(t) = \left(\overset{(n-1)}{\mathbf{Rem}_y} + d_n\right) e^{-\kappa_a(t-t_{n-1})}; \qquad t \in I_n \quad \text{(multiplicity condition)} \end{aligned} \tag{43}$$

- Using the previous solution, it follows that

$$\begin{aligned} &\frac{d\overset{(n)}{x}}{dt} = \kappa_a \cdot \gamma \overset{(n)}{y}(t) - \kappa_e \overset{(n)}{x} \Longrightarrow \frac{d\overset{(n)}{x}}{dt} + \kappa_e \overset{(n)}{x} = \kappa_a \cdot \gamma \overset{(n)}{y}(t) \\ &\frac{d}{dt}\left[e^{\kappa_e t}\right] = e^{\kappa_e t}\kappa_a \cdot \gamma \overset{(n)}{y}(t) \Longrightarrow \int d\left[e^{\kappa_e} \overset{(n)}{x}(t)\right] = \int e^{\kappa_e}\kappa_a \cdot \gamma \left(\overset{(n-1)}{\mathbf{Rem}_y}\right) e^{-\kappa_a(t-t_{n-1})}dt \\ &\Longrightarrow \overset{(n)}{x}(t) = \kappa_a \cdot \gamma \left(\overset{(n-1)}{\mathbf{Rem}_y}\right) e^{\kappa_a t_{n-1}} \left[\frac{e^{-\kappa_a t}}{\kappa_a - \kappa_e} + Ce^{-\kappa_e t}\right] \end{aligned} \tag{44}$$

Finally, using the initial conditions to find the value of the integration constant $C$ and the multiplicity conditions, we can establish the desired result,

$$\overset{(n)}{x}(t) = \left(\frac{\kappa_a \cdot \gamma \cdot (\overset{(n-1)}{\mathbf{Rem}_y} + d_n)}{\kappa_a - \kappa_e} + \overset{(n-1)}{\mathbf{Rem}_x}\right) e^{-\kappa_e(t-t_{n-1})} - \left(\frac{\kappa_a \cdot \gamma \cdot (\overset{(n-1)}{\mathbf{Rem}_y} + d_n)}{\kappa_a - \kappa_e}\right) e^{-\kappa_a(t-t_{n-1})} \tag{45}$$

■

Next, we find explicit formulas for the sequences of the remainders

**Proposition 19** (Formulas for the Remainders)**.** *We refer to the amount of drug that remains in the intestinal tract,* $\overset{(n)}{y}(t_n)$*, and the concentration of the drug that still exists in the bloodstream,* $\overset{(n)}{x}(t_n)$*, at the end of the n-th interval or period between each dose,* $I_n = [t_{n-1}, t_n]$*. We denote these as* $\overset{(n)}{\boldsymbol{Rem}_y}$ *and* $\overset{(n)}{\boldsymbol{Rem}_x}$ *respectively. The following expressions represent these quantities:*

$$\overset{(n)}{\boldsymbol{Rem}_y} = \overset{(n)}{y}(t_n) = \sum_{i=1}^{n}\prod_{j=i}^{n} d_i\alpha_j; \qquad \alpha_s = e^{-\kappa_a t_s}, \qquad s = 1,2,3,\ldots,n \geq 1$$
$$\overset{(n)}{\boldsymbol{Rem}_x} = \overset{(n)}{x}(t_n) = \frac{\kappa_a\cdot\gamma}{\kappa_a-\kappa_e}\left[\sum_{i=1}^{n}\prod_{j=i}^{n} d_i\beta_j - \sum_{i=1}^{n}\prod_{j=i}^{n} d_i\alpha_j\right]; \alpha_s = e^{-\kappa_a\tau_s},\ \beta_s = e^{-\kappa_e\tau_s},\ s = 1,2,3,\ldots,n \geq 1$$
$$\overset{(0)}{\boldsymbol{Rem}_x} = \overset{(0)}{\boldsymbol{Rem}_y} = 0 \tag{46}$$

*Proof.* By Proposition 18, we know that the general solution can be expressed using the recurrence formula:

$$\begin{aligned}
\overset{(n)}{y}(t) &= \left(\overset{(n-1)}{\mathbf{Rem}_y} + d_n\right) e^{-\kappa_a(t-t_{n-1})}, \quad t\in[t_{n-1},t_n], \quad \tau_n = t_n - t_{n-1}\\
\overset{(n)}{x}(t) &= C_1 e^{-\kappa_e(t-t_{n-1})} - C_2 e^{-\kappa_a(t-t_{n-1})} \quad t\in[t_{n-1},t_n], \quad \tau_n = t_n - t_{n-1}\\
C_1 &= \frac{\kappa_a\cdot\gamma}{\kappa_a-\kappa_e}\left(\overset{(n-1)}{\mathbf{Rem}_y} + d_n\right) + \overset{(n-1)}{\mathbf{Rem}_x}\\
C_2 &= \frac{\kappa_a\cdot\gamma}{\kappa_a-\kappa_e}\left(\overset{(n-1)}{\mathbf{Rem}_y} + d_n\right)
\end{aligned} \tag{47}$$

- The proof of these formulas can be found using mathematical induction. Although we will not demonstrate the assertion in this manner, we will outline the steps of the recurrence until the behavior pattern becomes evident.

$$\begin{aligned}
\overset{(0)}{\mathbf{Rem}_y} &= \overset{(0)}{y}(0) = 0\\
\overset{(1)}{\mathbf{Rem}_y} &= \overset{(1)}{y}(t_1) = d_1\,\alpha_1\\
\overset{(2)}{\mathbf{Rem}_y} &= \left(\overset{(1)}{\mathbf{Rem}_y} + d_2\right) e^{-\kappa_a(t_2-t_1)}\\
\overset{(2)}{\mathbf{Rem}_y} &= (d_1\alpha_1 + d_2)\,\alpha_2\\
\overset{(2)}{\mathbf{Rem}_y} &= d_1\alpha_1\alpha_2 + d_2\alpha_2\\
\overset{(3)}{\mathbf{Rem}_y} &= (d_1\alpha_1\alpha_2 + d_2\alpha_2 + d_3)\alpha_3\\
\overset{(3)}{\mathbf{Rem}_y} &= d_1\alpha_1\alpha_2\alpha_3 + d_2\alpha_2\alpha_3 + d_3\alpha_3\\
\overset{(4)}{\mathbf{Rem}_y} &= d_1\alpha_1\alpha_2\alpha_3\alpha_4 + d_2\alpha_2\alpha_3\alpha_4 + d_3\alpha_3\alpha_4 + d_4\alpha_4\\
&\vdots \quad \vdots\\
\overset{(n)}{\mathbf{Rem}_y} &= \sum_{i=1}^{n}\prod_{j=i}^{n} d_i\alpha_j
\end{aligned} \tag{48}$$

- For the remainder of $\overset{(n)}{x}(t)$:

$$
\begin{aligned}
\overset{(0)}{\mathbf{Rem}}_x &= \overset{(0)}{x}(0) = 0 \\
\overset{(1)}{\mathbf{Rem}}_x &= \overset{(1)}{x}(t_1) = C_1 e^{-\kappa_e \tau_1} - C_2 e^{-\kappa_a \tau_1} \\
\overset{(1)}{\mathbf{Rem}}_x &= \left[\frac{\kappa_a \cdot \gamma}{\kappa_a - \kappa_e}\left(\cancelto{0}{\overset{(0)}{\mathbf{Rem}}_y} + d_1\right) + \cancelto{0}{\overset{(0)}{\mathbf{Rem}}_x}\right] e^{-\kappa_e \tau_1} - \left[\frac{\kappa_a \cdot \gamma}{\kappa_a - \kappa_e}\left(\cancelto{0}{\overset{(0)}{\mathbf{Rem}}_y} + d_1\right)\right] e^{-\kappa_a \tau_1} \\
\overset{(1)}{\mathbf{Rem}}_x &= \frac{\kappa_a \cdot \gamma}{\kappa_a - \kappa_e}\left(d_1\beta_1 - d_1\alpha_1\right) \\
\overset{(2)}{\mathbf{Rem}}_x &= \overset{(2)}{x}(t_2) = C_1 e^{-\kappa_e \tau_2} - C_2 e^{-\kappa_a \tau_2} \\
\overset{(2)}{\mathbf{Rem}}_x &= \left[\frac{\kappa_a \cdot \gamma}{\kappa_a - \kappa_e}\left(\overset{(1)}{\mathbf{Rem}}_y + d_2\right) + \overset{(1)}{\mathbf{Rem}}_x\right] e^{-\kappa_e \tau_2} - \left[\frac{\kappa_a \cdot \gamma}{\kappa_a - \kappa_e}\left(\overset{(1)}{\mathbf{Rem}}_y + d_2\right)\right] e^{-\kappa_a \tau_2} \\
\overset{(2)}{\mathbf{Rem}}_x &= \frac{\kappa_a \cdot \gamma}{\kappa_a - \kappa_e}\left[(d_1\beta_1\beta_2 + d_2\beta_2) - (d_1\alpha_1\alpha_2 + d_2\alpha_2)\right] \\
\overset{(3)}{\mathbf{Rem}}_x &= \left[\frac{\kappa_a \cdot \gamma}{\kappa_a - \kappa_e}\left(\overset{(2)}{\mathbf{Rem}}_y + d_3\right) + \overset{(2)}{\mathbf{Rem}}_x\right] e^{-\kappa_e \tau_3} - \left[\frac{\kappa_a \cdot \gamma}{\kappa_a - \kappa_e}\left(\overset{(2)}{\mathbf{Rem}}_y + d_3\right)\right] e^{-\kappa_a \tau_3} \\
\overset{(3)}{\mathbf{Rem}}_x &= \frac{\kappa_a \cdot \gamma}{\kappa_a - \kappa_e}\left[(d_1\beta_1\beta_2\beta_3 + d_2\beta_2\beta_3 + d_3\beta_3) - (d_1\alpha_1\alpha_2\alpha_3 + d_2\alpha_2\alpha_3 + d_3\alpha_3)\right] \\
&\vdots \qquad\qquad \vdots \\
\overset{(n)}{\mathbf{Rem}}_x &= \frac{\kappa_a \cdot \gamma}{\kappa_a - \kappa_e}\left[\sum_{i=1}^{n}\prod_{j=i}^{n} d_i\beta_j - \sum_{i=1}^{n}\prod_{j=i}^{n} d_i\alpha_j\right]
\end{aligned}
\tag{49}
$$

■

■

## B.3 Proof of Proposition 4

*Proof.* Since the solution function $\overset{(n)}{x}(t)$ in the time interval $I_n = [(n-1)\tau, n\tau]$ satisfies the usual Fundamental Theorem of Calculus, we can find a primitive or antiderivative $\overset{(n)}{x}(t)$ of the solution to the initial value problem in the theorem, $\overset{(n)}{x}(t)$, and then apply the Fundamental Theorem of Calculus.

■

## B.4 Proof of Proposition 5

*Proof.* Observe that

$$
\begin{aligned}
AUC_{[0,\infty]} &= \lim_{t\to\infty}\int_0^t \overset{(1)}{x}(u)\,du \\
&= \lim_{t\to\infty}\int_0^t \left(C_1 e^{-\kappa_e u} - C_2 e^{-\kappa_a u}\right)du; \qquad C_1 = C_2 = \frac{\kappa_a\,\gamma\,d}{\kappa_a - \kappa_e} \\
&= C_1 \lim_{t\to\infty}\left(\frac{1}{\kappa_e} - e^{-\kappa_e\,t} - \frac{1}{\kappa_a} + e^{-\kappa_a\,t}\right) \\
&= \frac{\kappa_a\,\gamma\,d}{\kappa_a - \kappa_e}\left(\frac{1}{\kappa_e} - \frac{1}{\kappa_a}\right)
\end{aligned}
\tag{50}
$$

■

### B.5 Proof of Proposition 6

*Proof.* Since the solution given by Theorem (2) is smooth in the usual calculus sense, we only need to verify that the Hilger derivative at the endpoint $(n-1)\tau$ is positive.

Without loss of generality, let $\kappa_a > \kappa_e$ as the proof for $\kappa_e > \kappa_a$ is similar due to the symmetry of the solution concerning these two parameters.

$$\begin{gathered}\kappa_a > \kappa_e \implies \beta > \alpha \implies \kappa_a\beta > \kappa_e\alpha \implies \kappa_a - \kappa_e > \kappa_a\beta - \kappa_e\alpha \\ \kappa_a(1-\beta) > \kappa_e(1-\alpha) \implies \frac{\kappa_a(1-\beta)}{\kappa_e(1-\alpha)} > 1\end{gathered} \tag{51}$$

With the previous result, we have proven that $C_2 > C_1$. Therefore, $\overset{(n)}{t}_{\max} > 0$ and $\overset{(n)\triangle}{x}((n-1)\tau) > 0$.

Lastly, we find the critical points for each period and verify using the second derivative criterion that at $\overset{(n)}{t}_{\max}$, the plasma concentration $\overset{(n)}{x}(t)$ reaches its maximum value. ∎

### B.6 Proof of Theorem 8

*Proof.* Notice that for a fixed $t \in I_n$ and $n$:

$$\begin{aligned}\left|\overset{(n)}{x}(t) - \overset{(n-1)}{x}(t-\tau)\right| &= \left|\beta^n\left(\frac{\kappa_a\,\gamma\,d}{\kappa_a-\kappa_e}\right)e^{-\kappa_e(t-n\tau)} - \alpha^n\left(\frac{\kappa_a\,\gamma\,d}{\kappa_a-\kappa_e}\right)e^{-\kappa_a(t-n\tau)}\right| \\ &= \left(\frac{\kappa_a\,\gamma\,d}{V|\kappa_a-\kappa_e|}\right)\left|\beta^n e^{-\kappa_e(t-n\tau)} - \alpha^n e^{-\kappa_a(t-n\tau)}\right| \\ &\le \left(\frac{\kappa_a\,\gamma\,d}{V|\kappa_a-\kappa_e|}\right)(\beta^n + \alpha^n)\end{aligned}$$

Hence,

$$\sup_{t\in I_n}\left|\overset{(n)}{x}(t) - \overset{(n-1)}{x}(t-\tau)\right| \le \left(\frac{\kappa_a\,\gamma\,d}{V|\kappa_a-\kappa_e|}\right)(\beta^n + \alpha^n)$$

So that

$$0 \le \lim_{n\to\infty}\left[\sup_{t\in I_n}\left|\overset{(n)}{x}(t) - \overset{(n-1)}{x}(t-\tau)\right|\right] \le \lim_{n\to\infty}\left(\frac{\kappa_a\,\gamma\,d}{V|\kappa_a-\kappa_e|}\right)(\beta^n + \alpha^n) = 0$$

Thus, by the squeeze theorem:

$$\lim_{n\to\infty}\left[\sup_{t\in I_n}\left|\overset{(n)}{x}(t) - \overset{(n-1)}{x}(t-\tau)\right|\right] = 0$$

∎

### B.7 Proof of Proposition 9

*Proof.* We will use the result from the previous proposition and the definition of steady state. In the steady state, the constants satisfy:

$$C_1 = \frac{\kappa_a d\gamma}{\kappa_a - \kappa_e}\frac{1}{1-\beta}; \quad C_2 = \frac{\kappa_a d\gamma}{\kappa_a - \kappa_e}\frac{1}{1-\alpha} \tag{52}$$

Therefore, $\frac{C_2}{C_1} = \frac{1-\beta}{1-\alpha}$. By substituting these values into the result of the previous proposition, we obtain the desired result. Finally, to demonstrate that this quantity is positive, we can use the chain of implications used earlier: $\kappa_a > \kappa_e$ implies that $\alpha < \beta$, which further implies that $C_2 > C_1$.

∎

### B.8 Proof of Theorem 10.

*Proof.* To prove the main statement, we begin proving the following preliminary proposition

**Proposition 20.** *Consider the following short-hand notations:*

$$\begin{aligned} p_1 &= -\frac{\kappa_a}{\kappa_a - \kappa_e}, \qquad p_2 = -\frac{\kappa_e}{\kappa_a - \kappa_e}, \\ p_3 &= \left(\frac{\kappa_a}{\kappa_e}\right)^{p_1}, \qquad p_4 = \left(\frac{\kappa_a}{\kappa_e}\right)^{p_2}, \\ z &= 1 - \alpha, \qquad w = 1 - \beta. \end{aligned} \tag{53}$$

*If $\kappa_a > \kappa_e > 0$, then it follows that*

$$\begin{aligned} & p_2 > p_1, \qquad p_4 > p_3, \qquad p_2 - p_1 = 1 \implies \begin{cases} p_1 = p_2 - 1 \\ p_2 = p_1 + 1 \end{cases} \\ & \beta > \alpha > 0 \implies z > w > 0 \implies \begin{cases} z' = \frac{dz}{d\tau} = \kappa_a \alpha \\ w' = \frac{dw}{d\tau} = \kappa_e \beta \end{cases} \\ & \textit{and} \quad \frac{w^{p_1}}{z^{P_2}} > 0 \end{aligned} \tag{54}$$

*Proof.* Direct calculations based on the hypothesis and definition of new variables.

■

By substituting the new notation defined in Proposition 20 and simplifying, the formulas for the minimum and maximum plasma concentrations in the steady-state, $\underline{SS}$ and $\overline{SS}$, are as follows:

$$\begin{aligned} \underline{SS}(d,\tau) &= \frac{\kappa_a\, d\gamma}{\kappa_a - \kappa_e}\left[\left(\frac{\beta}{1-\beta}\right) - \left(\frac{\alpha}{1-\alpha}\right)\right] \\ \underline{SS}(d,\tau) &= \frac{\kappa_a\, d\gamma}{\kappa_a - \kappa_e}\left[\frac{\beta - \alpha}{(1-\alpha)(1-\beta)}\right] \\ \overline{SS}(d,\tau) &= \frac{\kappa_a\, d\gamma}{\kappa_a - \kappa_e}\left[\frac{1}{1-\beta}\left(\frac{\kappa_a(1-\beta)}{\kappa_e(1-\alpha)}\right)^{-\frac{\kappa_e}{\kappa_a - \kappa_e}} - \frac{1}{1-\alpha}\left(\frac{\kappa_a(1-\beta)}{\kappa_e(1-\alpha)}\right)^{-\frac{\kappa_a}{\kappa_a - \kappa_e}}\right] \\ \overline{SS}(d,\tau) &= \frac{\kappa_a\, d\gamma}{\kappa_a - \kappa_e}\left(\frac{w^{p_1}}{z^{p_2}}\right)(p_4 - p_3) \end{aligned}$$

(a) Note that both functions are positive because the factors composing them are positive. Additionally, observe that both functions share the same first factor, which is directly proportional to $d$. Therefore, both partial derivatives of the mentioned functions with respect to $d$ are positive, indicating that both functions are increasing with respect to $d$.

(b) By taking partial derivatives with respect to $\tau$ of the expressions obtained in the previous item, we have:

$$\begin{aligned} \frac{\partial}{\partial\tau}\underline{SS}(d,\tau) &= A\left[\frac{\kappa_a\alpha}{(1-\alpha)^2} - \frac{\kappa_e\beta}{(1-\beta)^2}\right] \\ \frac{\partial}{\partial\tau}\overline{SS}(d,\tau) &= \frac{\partial\overline{SS}}{\partial\tau} = A(p_4 - p_3)\left(\frac{w^{p_1}}{z^{p_2}}\right)\left(\frac{\kappa_a\kappa_e}{\kappa_a - \kappa_e}\right)\left[\frac{\alpha - \beta}{(1-\alpha)(1-\beta)}\right] \end{aligned} \tag{55}$$

Using the results from Proposition 20, it is evident that the function $\overline{SS}$ is decreasing with respect to $\tau$.

To demonstrate that the function $\underline{SS}$ is also decreasing with respect to $\tau$, we define a new function $f(x)$ in such a way that the second factor can be rewritten as $f(a) - f(b)$,

$$f(x) := \frac{xe^{-x\tau}}{(1-e^{-x\tau})^2} \quad \text{for} \quad x > 0, \quad \tau > 0.$$
$$f(\kappa_a) = \frac{\kappa_a \alpha}{(1-\alpha)^2}, \qquad f(\kappa_e) = \frac{\kappa_e \beta}{(1-\beta)^2} \tag{56}$$

The function $f(x)$ defined above has a derivative given by,

$$f'(x) = \frac{e^{\tau x}\left[(1-\tau x)e^{\tau x} - (1+\tau x)\right]}{(e^{\tau x}-1)^3} = \frac{e^{\tau x}\left[h_1(x) - h_2(x)\right]}{(e^{\tau x}-1)^3} \tag{57}$$

Note that $f'(x)$ is negative because $h_1(x) = (1+\tau x)e^{\tau x} < h_2(x) = (1+\tau x)$ due to $\lim_{x\to 0} h_1(x) = \lim_{x\to 0} h_2(x) = 1$ and additionally, $h_2{}'(x) = \tau > 0$ and $h_1{}'(x) = -\tau^2\, xe^{\tau x} < 0$ for all $x > 0$.

This implies that $f(x)$ is decreasing in the interval $x > 0$. Thus, for $\kappa_a > \kappa_e > 0$ and $\tau > 0$, we can conclude that $p_5 = (\kappa_a\, \alpha)/(1-\alpha)^2 < p_6 = (\kappa_e\, \beta)/(1-\beta)^2$. Thus, $\underline{SS}$ is decreasing with respect to $\tau$.

■

## B.9 Proof of Theorem 11

*Proof.* Let $\ell = \overline{SS} - \underline{SS}$. By using the definitions and results from Proposition 20, we have:

$$\ell = \overline{SS} - \underline{SS} = \frac{\kappa_a\, d\, \gamma}{\kappa_a - \kappa_e}\left[(p_4 - p_3)\left(\frac{w^{p_1}}{z^{p_2}}\right) + \left(\frac{z-w}{z\, w}\right)\right] \tag{58}$$

Note that $\ell > 0$ for all $\kappa_a > \kappa_e > 0$, $d > 0$, and $\tau > 0$.
To prove the second part, we again use the definitions from Proposition 20. Note that if $\tau \to \infty$, then $\alpha \to 0$, $\beta \to 0$, $z \to 1$, and $w \to 1$. We obtain the desired result by substituting the original variables $p_i$, $w$, and $z$.

■

## B.10 Proof of Proposition 12

*Proof.* From the definition of steady state, it follows that

$$\text{AUC}^{s.s.} = \lim_{n\to\infty} \text{AUC}_{I_n} \tag{59}$$

Under the assumption that $\kappa_a > \kappa_e > 0$, $\alpha = e^{-\kappa_a \tau}$ and $\beta = e^{-\kappa_e \tau}$, then $0 < \alpha < \beta < 1$ from which it can be contended that,

$$\lim_{n\to\infty} \alpha^n = \lim_{n\to\infty} \beta^n = 0 \tag{60}$$

Now, following Proposition 4 and Proposition 5 we can conclude that,

$$\text{AUC}^{s.s.} = \lim_{n\to\infty} \frac{\kappa_a\, \gamma\, d}{\kappa_a - \kappa_e}\left[\left(\frac{1-\beta^n}{\kappa_e}\right) - \left(\frac{1-\alpha^n}{\kappa_a}\right)\right] = \text{AUC}_{[0,\infty)} \tag{61}$$

■

## B.11 Proof of Theorem 13

*Proof.* For any $\bar{R} > \underline{R} > 0$, we can choose target levels $(\underline{SS}^*, \overline{SS}^*)$ such that $\underline{R} < \underline{SS}^* < \overline{SS}^* < \overline{R}$. We know establish the solutions to the system of nonlinear equations given by,

$$\begin{aligned} \underline{SS} &= \underline{SS}(\kappa_a, \kappa_e, \gamma, V, d, \tau) \\ \overline{SS} &= \overline{SS}(\kappa_a, \kappa_e, \gamma, V, d, \tau) \end{aligned} \tag{62}$$

Furthermore, for a fixed vector of physiological parameters $(\kappa_a, \kappa_e, \gamma)$, we can further view this problem as

$$\begin{aligned} \underline{SS} &= \underline{SS}(d, \tau) \\ \overline{SS} &= \overline{SS}(d, \tau) \end{aligned} \tag{63}$$

Within this logical framework, we must choose a pair $(d^*, \tau^*)$ such that

$$\underline{SS}(d^*, \tau^*) = \frac{\kappa_a\, d\, \gamma}{\kappa_a - \kappa_e} \left[ \left( \frac{\beta(\tau)}{1-\beta(\tau)} \right) - \left( \frac{\alpha(\tau)}{1-\alpha(\tau)} \right) \right] = \frac{\kappa_a\, d\, \gamma}{\kappa_a - \kappa_e} \Psi(\tau) = \underline{SS}^*$$

Hence, all solutions must satisfy

$$d^*(\tau) = \frac{\kappa_a - \kappa_e}{\kappa_a\, \gamma} \frac{\underline{SS}^*}{\Psi(\tau)}$$

Restricted to the curve, the upper limit can be seen as a function of $\tau$ as follows

$$\begin{aligned} \overline{\text{SS}}(d^*(\tau), \tau) &= \frac{\kappa_a d^*(\tau)\gamma}{\kappa_a - \kappa_e} \left[ \frac{1}{1-\beta} \left( \frac{\kappa_a(1-\beta)}{\kappa_e(1-\alpha)} \right)^{-\frac{\kappa_e}{\kappa_a - \kappa_e}} - \frac{1}{1-\alpha} \left( \frac{\kappa_a(1-\beta)}{\kappa_e(1-\alpha)} \right)^{-\frac{\kappa_a}{\kappa_a - \kappa_e}} \right] \\ &= \frac{\kappa_a d^*(\tau)\gamma}{\kappa_a - \kappa_e} \Phi(\tau) \\ &= \frac{\Phi(\tau)}{\Psi(\tau)} \underline{SS}^* \end{aligned}$$

Thus, it suffices to show there is a $\tau > 0$ satisfying the equation

$$\overline{\text{SS}}(d^*(\tau), \tau) = \frac{\Phi(\tau)}{\Psi(\tau)} \underline{SS}^* = \overline{SS}^*$$

Equivalently, the question boils down to determining

$$\frac{\overline{SS}^*}{\underline{SS}^*} \overset{?}{\in} \text{Range}(f(\tau)); \quad \text{where } f(\tau) = \frac{\Phi(\tau)}{\Psi(\tau)}$$

**Proposition 21** (Range of the quotient)**.** *Let $\kappa_a > \kappa_e$. Then the function $f(\tau)$ defined as*

$$\begin{aligned} f(\tau) &= \frac{\Phi(\tau; \kappa_a, \kappa_e, \gamma, V)}{\Psi(\tau; \kappa_a, \kappa_e, \gamma, V)} \\ \Psi(\tau) &= \frac{\beta}{1-\beta} - \frac{\alpha}{1-\alpha} \\ \Phi(\tau) &= \frac{1}{1-\beta} \left( \frac{\kappa_a(1-\beta)}{\kappa_e(1-\alpha)} \right)^{p_2} - \frac{1}{1-\alpha} \left( \frac{\kappa_a(1-\beta)}{\kappa_e(1-\alpha)} \right)^{p_1} \\ \textit{where} \quad \alpha &= e^{-\kappa_a \tau} \qquad \beta = e^{-\kappa_e \tau} \qquad p_1 = -\frac{\kappa_a}{\kappa_a - \kappa_e} \qquad p_2 = -\frac{\kappa_e}{\kappa_a - \kappa_e} \end{aligned} \tag{64}$$

*is well-defined in the domain $(0, \infty)$, has a range of $(1, \infty)$ and is a continuous function.*

*Proof.* To demonstrate that the range of $f(\tau)$is $(1, \infty)$ in the domain $(0, \infty)$, we will consider the following auxiliary functions:

$$\begin{aligned}
g(\tau) &= \frac{\kappa_a(1-\beta)}{\kappa_e(1-\alpha)} \\
f_1(\tau) &= \frac{1}{\Psi(\tau)}\left(\frac{1}{1-\alpha}\right) g(\tau)^{p_1} = \left(\frac{1-\beta}{\beta-\alpha}\right) f(\tau)^{p_1} \\
f_2(\tau) &= \frac{1}{\Psi(\tau)}\left(\frac{1}{1-\beta}\right) g(\tau)^{p_2} = \left(\frac{1-\alpha}{\beta-\alpha}\right) f(\tau)^{p_2} \\
f(\tau) &= f_2(\tau) - f_1(\tau)
\end{aligned} \tag{65}$$

a) Following L'Hôpital's rule, we have:

$$\lim_{\tau\to 0} g(\tau) = \left(\frac{\kappa_a}{\kappa_e}\right) \lim_{\tau\to 0}\left(\frac{1-\beta}{1-\alpha}\right) = \left(\frac{\kappa_a}{\kappa_e}\right) \lim_{\tau\to 0}\left(\frac{\kappa_e\beta}{\kappa_a\alpha}\right) = 1 \tag{66}$$

Now,

$$\lim_{\tau\to 0} f_2(\tau) = \lim_{\tau\to 0}\left(\frac{1-\alpha}{\beta-\alpha}\right)\left(\lim_{\tau\to 0} f(\tau)\right)^{p_2} = \lim_{\tau\to 0}\left(\frac{\kappa_a\alpha}{\kappa_e\beta-\kappa_a\alpha}\right)\left(\lim_{\tau\to 0} f(\tau)\right)^{p_2} = -p_1 \tag{67}$$

and similarly,

$$\lim_{\tau\to 0} f_1(\tau) = \lim_{\tau\to 0}\left(\frac{1-\beta}{\beta-\alpha}\right)\left(\lim_{\tau\to 0} f(\tau)\right)^{p_1} = \lim_{\tau\to 0}\left(\frac{\kappa_e\beta}{\kappa_e\beta-\kappa_a\alpha}\right)\left(\lim_{\tau\to 0} f(\tau)\right)^{p_1} = -p_2 \tag{68}$$

Therefore,

$$\lim_{\tau\to 0} f(\tau) = \lim_{\tau\to 0} f_1(\tau) - \lim_{\tau\to 0} f_2(\tau) = p_2 - p_1 = 1 \tag{69}$$

b) Since $f(\tau) = \dfrac{\Phi(\tau)}{\Psi(\tau)}$, we have:

$$\begin{aligned}
&\lim_{\tau\to\infty} \Psi(\tau) = 0 \\
&\lim_{\tau\to\infty} \Phi(\tau) = \left(\frac{\kappa_a}{\kappa_e}\right)^{p_2} - \left(\frac{\kappa_a}{\kappa_e}\right)^{p_1} > 0 \qquad \text{because} \quad \kappa_a > \kappa_e \quad \text{and} \quad p_2 > p_1
\end{aligned} \tag{70}$$

Therefore,

$$\lim_{\tau\to\infty} f(\tau) = \frac{\lim_{\tau\to\infty} \Phi(\tau)}{\lim_{\tau\to\infty} \Psi(\tau)} = \infty \tag{71}$$

c) Because $f(\tau)$ is the ratio of analytic functions, $\Phi(\tau)$ and $\Psi(\tau)$, defined on $(0,\infty)$, then $f(\tau)$ is also continuous on $(0,\infty)$.

Given $\lim_{\tau\to 0} f(\tau) = 1$, $\lim_{\tau\to\infty} f(\tau) = \infty$ and $f(\tau)$ is continuous on $(0,\infty)$, it follows by the Intermediate Value theorem that the range of $f(\tau)$ is $(1,\infty)$.

■

Following Proposition 21, for any $(\underline{SS}^*, \overline{SS}^*)$ such that $\underline{R} < \underline{SS}^* < \overline{SS}^* < \overline{R}$, there exists $\tau^* = \tau^*(\underline{SS}^*, \overline{SS}^*)$ such that

$$f(\tau^*) = \frac{\overline{SS}^*}{\underline{SS}^*}$$

Thus the pair $(d^*, \tau^*) = (d^*(\tau^*(\underline{SS}^*, \overline{SS}^*)), \tau^*(\underline{SS}^*, \overline{SS}^*))$ is such that

$$\begin{aligned}
\underline{SS}^* &= \underline{SS}(d^*, \tau^*) \\
\overline{SS}^* &= \overline{SS}(d^*, \tau^*)
\end{aligned} \tag{72}$$

which means that $(d^*, \tau^*) \in \mathcal{E}(\underline{R}, \bar{R}; \kappa_a, \kappa_e, \gamma)$, so that $\mathcal{E}(\underline{R}, \bar{R}; \kappa_a, \kappa_e, \gamma) \neq \emptyset$.

■

Furthermore, we can prove the effective dose is unique for a given target level $(\underline{SS}^*, \overline{SS}^*)$, since the map $f(\tau)$ introduced above is also injective:

**Proposition 22** (The function $f(\tau)$ is strictly increasing)**.** *The function $f(\tau) : (0, \infty) \longrightarrow (1, \infty)$ is strictly increasing, i.e., $f(\tau)$ is bijective.*

*Proof.* In Theorem 21 we proved that $f(\tau)$, with a domain of $[0, \infty)$ and a range of $[1, \infty)$, is continuous and satisfies: $\lim_{\tau \to 0} f(\tau) = 1$ and $\lim_{\tau \to \infty} f(\tau) = \infty$. To prove that $f(\tau)$ is bijective, we will show that it is strictly increasing by arguing that its derivative is positive, $f'(\tau) > 0$, for all $\tau > 0$.

Departing from the definition of $f(\tau)$, we can derive the following simplified expression of its derivative:

$$f'(\tau) = \left[ \left( \frac{\kappa_a}{\kappa_e} \right)^{p_2} - \left( \frac{\kappa_a}{\kappa_e} \right)^{p_1} \right] \left[ \frac{(1-\beta)^{p_1}}{(1-\alpha)^{p_2}} \right] \left[ \frac{\kappa_e \beta (1-\alpha)^2}{(\beta-\alpha)^2} - \frac{\kappa_a \alpha (1-\beta)^2}{(\beta-\alpha)^2} - \frac{\kappa_a \, \kappa_e}{\kappa_a - \kappa_e} \right] \tag{73}$$

Note that the first two of the three factors that compose the derivative are positive. It remains to prove that the third factor is also positive. To analyze the third factor more easily, we will use the following notation: let $a = \kappa_a$, $b = \kappa_e$, $z = 1-\alpha$, $w = 1-\beta$. Since $a > b > 0$ and $\tau > 0$, we have $0 < w < z < 1$. With this new notation, the derivative can be expressed as:

$$\begin{aligned} f'(\tau) &= \left[ \left( \frac{a}{b} \right)^{p_2} - \left( \frac{a}{b} \right)^{p_1} \right] \left[ \frac{w^{p_1}}{z^{p_2}} \right] \left[ \frac{a^2 w^2 z - a^2 w^2 - abw^2 z - abwz^2 + 2abwz + b^2 wz^2 - b^2 z^2}{(a-b)(z-w)^2} \right] \\ f'(\tau) &= \left[ \left( \frac{a}{b} \right)^{p_2} - \left( \frac{a}{b} \right)^{p_1} \right] \left[ \frac{w^{p_1}}{z^{p_2}} \right] \left[ \frac{bz(1-w)(aw-bz) - aw(1-z)(aw-bz)}{(a-b)(z-w)^2} \right] \end{aligned} \tag{74}$$

Let's denote by num the numerator of the third factor of the derivative of $f(\tau)$:

$$\text{num} = bz(1-w)(aw-bz) - aw(1-z)(aw-bz) \tag{75}$$

Let's prove that $\text{num} = bz(1-w)(aw-bz) - aw(1-z)(aw-bz) > 0$. This can be accomplished in two steps:

**Step 1:** <u>Prove that $aw - bz > 0$:</u> Consider the function $g(x) = \dfrac{xe^{x\tau}}{e^{x\tau} - 1}$ and prove that $g(\cdot)$ is increasing. The derivative, $g'(x) = \dfrac{e^{x\tau}\left[e^{x\tau} - (1 + x\tau)\right]}{(e^{x\tau} - 1)^2}$, is positive because the second term in the numerator dominates (is greater than) the first term in the numerator. This implies that $g(a) > g(b)$, which implies that indeed $aw - bz > 0$.

**Step 2:** <u>Prove that $\text{num} = bz(1-w)(aw-bz) - aw(1-z)(aw-bz)$:</u>
Consider the function $h(x) = \dfrac{x}{e^{x\tau} - 1}$.
This new function is decreasing because its derivative is negative. This is because $h'(x) = \dfrac{e^{x\tau} - (1 + x\tau e^{x\tau})}{(e^{x\tau} - 1)^2}$, and the second term in the numerator dominates the first term in the numerator. Therefore, $h(a) < h(b)$, which implies that: $\text{num} = bz(1-w)(aw-bz) - aw(1-z)(aw-bz) > 0$.

Hence, we can conclude that for all $\tau > 0$ and for any choice of $\kappa_a > \kappa_e > 0$, the function $f(\tau)$ is strictly increasing on $[0, \infty)$ and thus also bijective.

■

An immediate corollary of Proposition 22 is that the effective doses can be locally expressed as functions of the biological parameters and the target concentration levels:

$$\tau^* = \tau^*(\underline{\mathrm{SS}}^*, \overline{\mathrm{SS}}^*, \kappa_a, \kappa_e, \gamma)$$
$$d^* = d^*(\underline{\mathrm{SS}}^*, \overline{\mathrm{SS}}^*, \kappa_a, \kappa_e, \gamma)$$

# C Appendix - Omitted proofs - Other applications

## C.1 Proof of Theorem 14

*Proof.* To establish the validity of the theorem, we prove an equivalent proposition

**Proposition 23.** *Given any arbitrary dosage schedule given by the sequence* $\{(\delta_n, \tau_n)\}_{n=1}^{\infty}$*, the solution to the system of dynamic equations presented in* (21) *is:*

$$\begin{aligned} x(t) = \sum_{n=1}^{\infty} \mathbb{1}[t \in I_n] \overset{(n)}{x}(t); \quad \overset{(n)}{x}(t) = \left( \overset{\mathbf{(n-1)}}{\mathbf{Rem_x}} + \delta_n \right) e^{-\kappa_e(t-t_{n-1})}, \quad \beta_s = e^{-\kappa_e \tau_s} \\ \textit{where} \\ \overset{\mathbf{(n)}}{\mathbf{Rem_x}} = \sum_{i=1}^{n} \prod_{j=i}^{n} \delta_i \beta_j \quad \textit{and} \quad \overset{\mathbf{(0)}}{\mathbf{Rem_x}} = 0 \end{aligned} \tag{76}$$

*Proof.* We will prove the theorem using mathematical induction.

(a) **Base case:** We verify that the formula holds for $n = 1$. Using the initial condition and the continuity condition, we have,

$$\begin{aligned} \overset{(1)}{x}(t) = C_0 e^{\kappa_e t}; \qquad t \in [0, t_1] \\ \implies C_0 = \delta_1 \quad \therefore \quad C_0 = \overset{\mathbf{(0)}}{\mathbf{Rem_x}} + \delta_1 \end{aligned} \tag{77}$$

(b) **Induction hypothesis:** We assume that the formula holds for $n = k$, that is,

$$\begin{aligned} \overset{(k)}{x}(t) = \left( \overset{\mathbf{(k-1)}}{\mathbf{Rem_x}} + \delta_k \right) e^{-\kappa_e(t-t_{k-1})}, \quad t \in [t_{k-1}, t_k] \\ \text{where} \\ \overset{\mathbf{(k)}}{\mathbf{Rem_x}} = \sum_{i=1}^{k} \prod_{j=i}^{k} \delta_i \beta_j \end{aligned} \tag{78}$$

(c) **Induction step:** Using the initial condition, the continuity condition, and the induction hypothesis, we have,

$$\begin{aligned} \overset{(k+1)}{x}(t) &= C_k e^{\kappa_e(t-t_k)}, \qquad t \in [t_k, t_{k+1}] \\ \implies C_k &= \overset{(k)}{x}(t_k) + \delta_{k+1} \\ &= \left( \overset{\mathbf{(k-1)}}{\mathbf{Rem_x}} + \delta_k \right) e^{-\kappa_e(t_k-t_{k-1})} + \delta_{k+1} \\ &= \left( \overset{\mathbf{(k-1)}}{\mathbf{Rem_x}} + \delta_k \right) \beta_k + \delta_{k+1} \\ &= \left( \sum_{i=1}^{k-1} \prod_{j=i}^{k-1} \delta_i \beta_j + \delta_k \right) \beta_k + \delta_{k+1} \\ C_k &= \sum_{i=1}^{k} \prod_{j=i}^{k} \delta_i \beta_j + \delta_{k+1} \\ \therefore \qquad \overset{(k+1)}{x}(t) &= \left( \overset{\mathbf{(k)}}{\mathbf{Rem_x}} + \delta_{k+1} \right) e^{-\kappa_e(t-t_k)}, \quad t \in [t_k, t_{k+1}] \end{aligned} \tag{79}$$

■

■

## C.2 Proof of Theorem 15

*Proof.* To establish the validity of the theorem, we prove an equivalent proposition

**Proposition 24.** *Given any arbitrary dosage schedule given by the sequence* $\{(\delta_n, \tau_n)\}_{n=1}^{\infty}$*, and a sequence of F.A.T.* $\{s_n\}_{n=1}^{\infty}$*, the solution to the system of dynamic equations presented in* (23) *is:*

$$\begin{cases} y(t) = \sum_{n=1}^{\infty} \mathbb{1}[t \in I_n^1] \overset{(n)}{y_1}(t), \quad \overset{(n)}{y_1}(t) = d_n e^{-\kappa_a(t-t_{n-1})} \\ x(t) = \sum_{n=1}^{\infty} \mathbb{1}[t \in I_n^1] \overset{(n)}{x_1}(t) + \mathbb{1}[t \in I_n^2] \overset{(n)}{x_2}(t); \quad \begin{cases} \overset{(n)}{x_1}(t) = C_1 e^{-\kappa_e(t-t_{n-1})} - C_2 e^{-\kappa_a(t-t_{n-1})} \\ \overset{(n)}{x_2}(t) = C_3 e^{-\kappa_e(t-s_n)} \end{cases} \\ \textit{where} \\ I_n^1 = [t_{n-1}, s_n], \textit{\textbf{(Assimilation Phase)}}; \quad I_n^2 = [s_n, t_n], \textit{\textbf{(Clearance Phase)}}; \quad n = 1, \dots, \infty \\ C_1 = \frac{\kappa_a \cdot \gamma}{\kappa_a - \kappa_e} d_n + \overset{(\mathbf{n-1})}{\mathbf{Rem_x^2}} \\ C_2 = \frac{\kappa_a \cdot \gamma}{\kappa_a - \kappa_e} d_n \\ C_3 = \overset{(\mathbf{n})}{\mathbf{Rem_x^1}} \\ \overset{(\mathbf{0})}{\mathbf{Rem_x^2}} = 0 \end{cases} \tag{80}$$

*where,*

$$\overset{(\mathbf{n})}{\mathbf{Rem_x^1}} = \frac{\kappa_a \cdot \gamma}{\kappa_a - \kappa_e}(B - A) \left[\sum_{i=1}^{n} \beta^{n-i} d_i\right] \quad n = 1, 2, \dots, \infty$$

$$\overset{(\mathbf{n})}{\mathbf{Rem_x^2}} = \frac{\beta}{B} \overset{(\mathbf{n})}{\mathbf{Rem_x^1}} \quad n = 1, \dots, \infty \tag{81}$$

$$\begin{aligned} &\alpha = e^{-\kappa_a \tau}, \quad 0 < \alpha < 1 \quad \beta = e^{-\kappa_e \tau}, \quad 0 < \beta < 1 \\ &A = e^{-\kappa_a \sigma}, \quad 0 < A < 1 \quad B = e^{-\kappa_e \sigma}, \quad 0 < B < 1 \\ &\alpha^n = e^{-\kappa_a t_n}, \quad \beta^n = e^{-\kappa_e t_n}, \quad \alpha^{n-1} A = e^{-\kappa_a s_n}, \quad \beta^{n-1} B = e^{-\kappa_e s_n}; \quad n = 1, 2, \dots, \infty \\ &\alpha^0 = \beta^0 = 1. \end{aligned}$$

*Proof.* We first proof a pair of propositions before dealing with the main result

**Proposition 25.** *The Initial Value Problem (IVP),*

$$\begin{cases} \left.\begin{aligned} y'(t) &= -\kappa_a y(t) \\ x'(t) &= \kappa_a \gamma y(t) - \kappa_e x(t) \end{aligned}\right\} \quad t \in [t_{n-1}, s_n] \\ \textit{\textbf{with initial conditions,}} \\ y(t_{n-1}) = y_{10} \\ x(t_{n-1}) = x_{10} \end{cases} \tag{82}$$

*has a unique solution given by,*

$$\begin{aligned} y(t) &= \left(\frac{\kappa_a \gamma}{\kappa_a - \kappa_e} y_{10}\right) e^{-\kappa_a (t - t_{n-1})} \\ x(t) &= \left(\frac{\kappa_a \gamma}{\kappa_a - \kappa_e} y_{10} + x_{10}\right) e^{-\kappa_e (t - t_{n-1})} - \left(\frac{\kappa_a \gamma}{\kappa_a - \kappa_e} y_{10}\right) e^{-\kappa_a (t - t_{n-1})} \end{aligned} \tag{83}$$

*Proof.* The matrix $A$ associated with this first-order linear system has eigenvalues and eigenvectors,

$$A = \begin{bmatrix} -\kappa_a & 0 \\ \kappa_a \gamma & -\kappa_e \end{bmatrix} \longrightarrow \begin{cases} \lambda_1 = -\kappa_a \to \xi_1 = \begin{bmatrix} \dfrac{\kappa_a - \kappa_e}{\kappa_a \gamma} \\ 1 \end{bmatrix} \\ \lambda_2 = -\kappa_e \to \xi_2 = \begin{bmatrix} 0 \\ 1 \end{bmatrix} \end{cases} \tag{84}$$

Thus, the solution is,

$$\begin{bmatrix} y(t) \\ x(t) \end{bmatrix} = C_1 \begin{bmatrix} \dfrac{\kappa_e - \kappa_a}{\kappa_a \gamma} \\ 1 \end{bmatrix} e^{-\kappa_a (t - t_{n-1})} + C_2 \begin{bmatrix} 0 \\ 1 \end{bmatrix} e^{-\kappa_e (t - t_{n-1})} \tag{85}$$

Replacing the initial conditions at $t = t_{n-1}$, we have,

$$\begin{aligned} y(t) &= \left(\frac{\kappa_a \gamma}{\kappa_a - \kappa_e} y_{10}\right) e^{-\kappa_a (t - t_{n-1})} \\ x(t) &= \left(\frac{\kappa_a \gamma}{\kappa_a - \kappa_e} y_{10} + x_{10}\right) e^{-\kappa_e (t - t_{n-1})} - \left(\frac{\kappa_a \gamma}{\kappa_a - \kappa_e} y_{10}\right) e^{-\kappa_a (t - t_{n-1})} \end{aligned} \tag{86}$$

∎

**Proposition 26.** *The Initial Value Problem (IVP),*

$$\begin{cases} \left.\begin{aligned} y'(t) &= 0 \\ x'(t) &= -\kappa_e x(t) \end{aligned}\right\} \quad t \in [s_n, t_n] \\ \textbf{\textit{with initial conditions,}} \\ y(s_n) = 0 \\ x(s_n) = x_{20} \end{cases} \tag{87}$$

*has a unique solution given by,*

$$\begin{aligned} y(t) &= 0 \\ x(t) &= x_{20} e^{-\kappa_e (t - s_n)} \end{aligned} \tag{88}$$

*Proof.* The matrix $A$ associated with this first-order linear system has eigenvalues and eigenvectors,

$$A = \begin{bmatrix} 0 & 0 \\ 0 & -\kappa_e \end{bmatrix} \longrightarrow \begin{cases} \lambda_1 = 0 \to \xi_1 = \begin{bmatrix} 1 \\ 0 \end{bmatrix} \\ \lambda_2 = -\kappa_e \to \xi_2 = \begin{bmatrix} 0 \\ 1 \end{bmatrix} \end{cases} \tag{89}$$

Thus, the solution is,

$$
\begin{bmatrix} y(t) \\ \\ x(t) \end{bmatrix} = C_1 \begin{bmatrix} 1 \\ \\ 0 \end{bmatrix} e^{-\kappa_a (t-s_n)} + C_2 \begin{bmatrix} 0 \\ \\ 1 \end{bmatrix} e^{-\kappa_e (t-s_n)} \tag{90}
$$

Replacing the initial conditions at $t = s_n$, we have,

$$
\begin{aligned}
y(t) &= \left( \frac{\kappa_a \gamma}{\kappa_a - \kappa_e} y_{10} \right) e^{-\kappa_a (t-t_{n-1})} \\
x(t) &= \left( \frac{\kappa_a \gamma}{\kappa_a - \kappa_e} y_{10} + x_{10} \right) e^{-\kappa_e (t-t_{n-1})} - \left( \frac{\kappa_a \gamma}{\kappa_a - \kappa_e} y_{10} \right) e^{-\kappa_a (t-t_{n-1})}
\end{aligned} \tag{91}
$$

■

Now we proceed with the main proof:

(a) To prove the formulas for the solutions given in (80), we proceed as follows:

- The formula for $\overset{(n)}{y}_1(t)$ is obtained by solving the associated equation for $y(t)$ (see Proposition 26) and using the first initial condition and the first multiplicity condition yields the result.

- It is clear that from the third multiplicity condition we have $\overset{(n)}{y}_0(t) = 0$, which is not explicitly written.

- By Proposition 25 and the second multiplicity condition, we have

$$
\begin{aligned}
C_1 &= \frac{\kappa_a \gamma}{\kappa_a - \kappa_e} y_{10} + x_{10} \\
C_2 &= \frac{\kappa_a \gamma}{\kappa_a - \kappa_e} y_{10}
\end{aligned} \tag{92}
$$

  But, $y_{10} = d_n$ and $x_{10} = \overset{(n)}{x}_1(t_{n-1}) = \overset{(n-1)}{x}_2(t) = \overset{(\mathbf{n-1})}{\mathbf{Rem}}{}_{\mathbf{x}}^{\mathbf{2}}$

- By the fourth multiplicity condition, we have

$$
x(t) = x_{20} e^{-\kappa_e (t-s_n)} \tag{93}
$$

  and Proposition 26 implies that $x_{20} = \overset{(n-1)}{x}_2(s_n) = \overset{(\mathbf{n})}{\mathbf{Rem}}{}_{\mathbf{x}}^{\mathbf{1}}$

(b) We now proceed to prove the formulas for the remains given in (81) as follows:

- To prove the second formula for the remains, we only use the definition,

$$
\begin{aligned}
\overset{(\mathbf{n})}{\mathbf{Rem}}{}_{\mathbf{x}}^{\mathbf{1}} = \overset{(n)}{x}_2(t_n) &= C_3 e^{-\kappa_e (t_n - s_n)} \\
&= \overset{(\mathbf{n})}{\mathbf{Rem}}{}_{\mathbf{x}}^{\mathbf{1}} \frac{e^{-\kappa_e t_n}}{e^{-\kappa_e s_n}} \\
&= \overset{(\mathbf{n})}{\mathbf{Rem}}{}_{\mathbf{x}}^{\mathbf{1}} \frac{\beta^n}{\beta^{n-1} B} = \overset{(\mathbf{n})}{\mathbf{Rem}}{}_{\mathbf{x}}^{\mathbf{1}} \frac{\beta}{B}
\end{aligned} \tag{94}
$$

- To prove the formula for the first remainder, we proceed using mathematical induction:

  - **Base case.** Verifying the formula for $n = 1$ is straightforward. It can be seen from Proposition 25 by replacing the initial conditions.

- **Induction hypothesis:** Suppose the formula is valid for $n = k$

$$\overset{(\mathbf{k})}{\mathbf{Rem}_{\mathbf{x}}^{\mathbf{1}}} = \frac{\kappa_a \cdot \gamma}{\kappa_a - \kappa_e}(B - A)\left[\sum_{i=1}^{k} \beta^{k-i}\, d_i\right] \tag{95}$$

- **Induction step:** We must demonstrate that the formula is valid for $n = k + 1$

$$\overset{(\mathbf{k+1})}{\mathbf{Rem}_{\mathbf{x}}^{\mathbf{1}}} = \frac{\kappa_a \cdot \gamma}{\kappa_a - \kappa_e}(B - A)\left[\sum_{i=1}^{k+1} \beta^{(k+1)-i}\, d_i\right] \tag{96}$$

Using the definition and Proposition 26, we have:

$$\begin{aligned}
\overset{(\mathbf{k+1})}{\mathbf{Rem}_{\mathbf{x}}^{\mathbf{1}}} &= \overset{(k+1)}{x_2}(s_{k+1}) \\
&= \left[\frac{\kappa_a \cdot \gamma}{\kappa_a - \kappa_e} d_{k+1} + \overset{(k)}{x_2}(t_k)\right] e^{-\kappa_e(s_{k+1}-t_k)} - \left[\frac{\kappa_a \cdot \gamma}{\kappa_a - \kappa_e} d_{k+1} e^{-\kappa_a(s_{k+1}-t_k)}\right] \\
&= \left[\frac{\kappa_a \cdot \gamma}{\kappa_a - \kappa_e} d_{k+1} + \overset{(k)}{x_2}(t_k)\right] \frac{\beta^k B}{\beta^k} - \left[\frac{\kappa_a \cdot \gamma}{\kappa_a - \kappa_e} d_{k+1}\right] \frac{\alpha^k A}{\alpha^k} \\
&= \frac{\kappa_a \cdot \gamma}{\kappa_a - \kappa_e}(B - A) d_{k+1} + \overset{(k)}{x_2}(t_n) B \\
&= \frac{\kappa_a \cdot \gamma}{\kappa_a - \kappa_e}(B - A) d_{k+1} + \overset{(\mathbf{k})}{\mathbf{Rem}_{\mathbf{x}}^{\mathbf{2}}}\, B \qquad (97) \\
&= \frac{\kappa_a \cdot \gamma}{\kappa_a - \kappa_e}(B - A) d_{k+1} + \overset{(\mathbf{k})}{\mathbf{Rem}_{\mathbf{x}}^{\mathbf{1}}}\, B\, \frac{\beta}{B} \\
&= \frac{\kappa_a \cdot \gamma}{\kappa_a - \kappa_e}(B - A) d_{k+1} + \beta\, \frac{\kappa_a \cdot \gamma}{\kappa_a - \kappa_e}(B - A)\left[\sum_{i=1}^{k} \beta^{k-i}\, d_i\right] \\
&= \frac{\kappa_a \cdot \gamma}{\kappa_a - \kappa_e}(B - A)\left[\sum_{i=1}^{k+1} \beta^{(k+1)-i}\, d_i\right]
\end{aligned}$$

■

■
